# Mass production of shaped particles through vortex ring freezing

**DOI:** 10.1038/ncomms12401

**Published:** 2016-08-04

**Authors:** Duo An, Alex Warning, Kenneth G. Yancey, Chun-Ti Chang, Vanessa R. Kern, Ashim K. Datta, Paul H. Steen, Dan Luo, Minglin Ma

**Affiliations:** 1 Department of Biological and Environmental Engineering, Cornell University, Ithaca, New York 14853, USA; 2 School of Chemical and Biomolecular Engineering, Cornell University, Ithaca, New York 14853, USA; 3 Kavli Institute at Cornell for Nanoscale Science, Cornell University, Ithaca, New York 14853, USA; 4 Suzhou Institute of Nano-Tech and Nano-Bionics, Chinese Academy of Sciences, Suzhou 215123, China

## Abstract

A vortex ring is a torus-shaped fluidic vortex. During its formation, the fluid experiences a rich variety of intriguing geometrical intermediates from spherical to toroidal. Here we show that these constantly changing intermediates can be ‘frozen' at controlled time points into particles with various unusual and unprecedented shapes. These novel vortex ring-derived particles, are mass-produced by employing a simple and inexpensive electrospraying technique, with their sizes well controlled from hundreds of microns to millimetres. Guided further by theoretical analyses and a laminar multiphase fluid flow simulation, we show that this freezing approach is applicable to a broad range of materials from organic polysaccharides to inorganic nanoparticles. We demonstrate the unique advantages of these vortex ring-derived particles in several applications including cell encapsulation, three-dimensional cell culture, and cell-free protein production. Moreover, compartmentalization and ordered-structures composed of these novel particles are all achieved, creating opportunities to engineer more sophisticated hierarchical materials.

## Abstract (web summary)

The evolution of vortex flow exhibits a rich spectrum of complicated geometries. Here, An *et al*. utilize these features to control and mass produce inorganic and organic particles via an electrospraying process, whereby the shape of vortex rings translates into particles during a freezing process.

## Graphical abstract

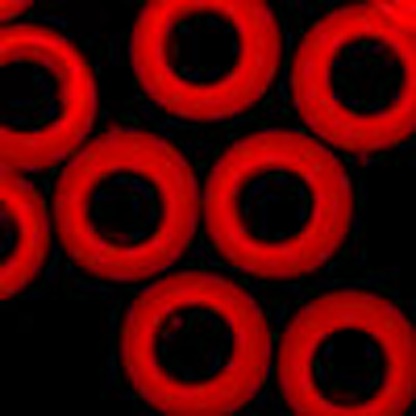

A vortex ring is a torus-shaped, fluidic region where the fluid spins around an imaginary axis line. Vortex rings exist almost ubiquitously in nature^1^,^2^,^3^,^4^ and have stimulated numerous studies for decades ranging from classical fluid mechanics^5^,^6^,^7^,^8^,^9^,^10^,^11^,^12^,^13^,^14^ to aquatic propulsion^15^,^16^, cardiac flows^17^,^18^,^19^, hydropropulsion/aeropropulsion^20^, micro jet thrusters^21^, and multi-scale stirring and mixing^22^. One simple way to generate a vortex ring is to have a droplet impacting the surface of a miscible liquid. When hitting the surface, the nearly spherical droplet deforms; during this deformation, many fluid intermediates with various intriguing, non-spherical shapes appear, including those resembling teardrops, jellyfishes, caps and donuts.

However, vortex rings rapidly evolve and are often short-lived^23^, making it almost impossible to harvest and utilize them as materials. In search of a novel material that can generate various, non-spherical shapes, we discover that a crosslinkable nanoclay system can be controlled to freeze vortex rings, creating a new class of vortex ring-derived particles (VRP) with uniform and sometimes unprecedented shapes including the teardrop-, jellyfish-, cap- and donut-shaped ones. Further adoption of a simple and inexpensive electrospraying technique enables the mass production of the VRP with controllable dimensions ranging from millimetres down to hundreds of microns (that is, microVRP). More importantly, we further show that it is possible to fabricate the microVRP from almost any materials as long as there exists a proper ‘freezing event', where the unstable liquid vortex rings are fixed into stable hydrogel or solid microparticles of a defined shape through a gelation or precipitation process. Theoretical analyses and a multiphase laminar fluid flow simulation also show that this freezing method, regardless what materials are used, can be a universal fabrication platform to make microVRP. Indeed, besides nanoclay, we are able to produce microVRP from several other very different materials, from polysaccharides (for example, alginate, chitosan) to silica nanocolloids, all by proper designs and control of the vortex ring freezing. Similarly, we can also fabricate hybrid microVRP from various material mixtures with functional components such as magnetic nanoparticles (NPs).

Among the different shapes of the microVRP, the donut (or toroidal) one is of special interests in the materials science field^24^,^25^,^26^. Compared with the more common spherical shapes, donut ones have several prominent advantages including always a higher surface/volume ratio, a shorter diffusion path within, and a better deformability. These advantages make the donut-shaped particles highly desirable in many applications such as catalytic reactions^27^, cell encapsulation^24^ and structural materials construction^28^. Several applications of these donut-microVRP are demonstrated including bioencapsulation, three-dimensional (3D) cell culture, and cell-free protein production. In addition, we show the successful fabrication of more complicated Janus and core–shell donut-microVRP by engineering the electrospray nozzle. The donut-microVRP can also be directionally and orderly organized in either linear or planar fashion by taking advantage of their unique geometry, paving the way for future assembly of more sophisticated hierarchical materials.

## Results

### Fabrication of VRP by freezing vortex rings

**Figure 1:**
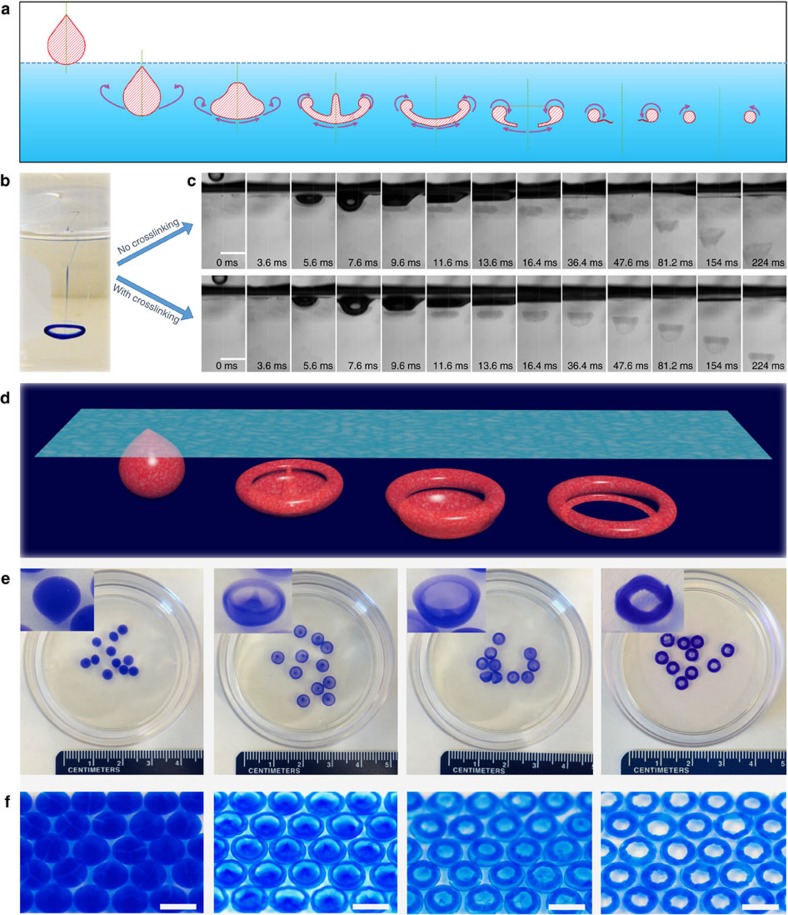
Droplet-formed vortex ring and VRP made by freezing vortex rings. (**a**) Schematic illustration of the vortex ring formation process (a water drop striking a water surface). (**b**) Digital image of a vortex ring formed by dripping a drop of ink into a water pool. (**c**) High-speed camera images of the vortex ring formation process by dripping a drop of nanoclay solution into a water pool (upper) and into a crosslinking buffer pool (lower). (**d**–**f**) Four typical VRP: teardrop-, jellyfish-, cap- and donut-ones. 3D illustration (**d**), digital images (**e**) and close-packed monolayers (**f**). Scale bars, 3 mm (**c**); 6 mm (**f**).

A simple way to form a vortex ring is to drop a droplet into a miscible liquid^9^,^28^. In a typical process, when a droplet hits the free surface of a miscible liquid at a sufficient impact speed, the droplet starts to deform in order to dissipate the energy by curling back (see Fig. 1a for the time sequence). As the edge continues to curl, the centre of the droplet becomes thinner and thinner. Eventually, when the centre is too thin to withstand the surface tension, it breaks and a donut shape is formed (Fig. 1b).

To more precisely visualize the vortex ring formation and investigate whether it is possible to utilize this process to fabricate novel particles, we need a material that allows controllable ‘freezing' of the vortex ring (Supplementary Note 1). We discover that a nanoclay system is ideal here because of its unique, controllable gelation properties. The nanoclay (Laponite XLG) possess a disk-like structure with a non-homogeneous charge distribution: negative charges on the disk surface and positive charges on the rim^29^. This special structure endows nanoclay with unique properties: in deionized water, the nanoclay disks are homogeneously distributed; whereas, in the presence of certain ionic species, the nanoclay disks pack together to generate a ‘house-of-cards' structure, forming a hydrogel^30^ (Supplementary Fig. 1a). Importantly, the viscosity (∼1.7 cP) and surface tension (∼72 mN m^−1^) of the nanoclay solution are well suitable for the formation of vortex rings within the crosslinking buffer, while the gelation speed can be precisely controlled. We used a high-speed camera to capture a series of images of vortex rings formed by a drop of nanoclay solution impacting an aqueous bath, with or without gelation reaction (Fig. 1c). In case of no gelation (that is, in a deionized water), the droplet moves along while spreading out with the front edges curling back. The shape of the droplet is constantly changing, experiencing in order, a teardrop-, jellyfish-, cap- and donut-shape. This whole process lasted for merely 100 ms, and the nanoclay solution finally dispersed homogeneously (see the upper row of Fig. 1c). In contrast, in the presence of gelation (that is, in a 100 mM CaCl_2_ solution), the vortex ring maintains its shape (that is, formed a VRP) starting from 36 ms (see the bottom row of Fig. 1c). As a result, by tuning the viscosity and impact speed of the droplet, we successfully captured the intermediates and produced VRP with various intriguing shapes (Fig. 1d,e). Some of these VRP may be difficult to fabricate using other traditional methods such as soft lithography^27^,^31^, microfluidics^32^ or self-assembly^33^. The formation of each of the VRP was also captured *in situ* and in real time by the high-speed camera images (Supplementary Fig. 2). Moreover, their unique shapes allow us to assemble the VRP in an ordered fashion, for example, into an orientated, close-packed monolayer via a strategy similar to a Langmuir–Blodgett deposition (Fig. 1f; Supplementary Fig. 3; Supplementary Note 2).

### Mass production of microVRP

**Figure 2:**
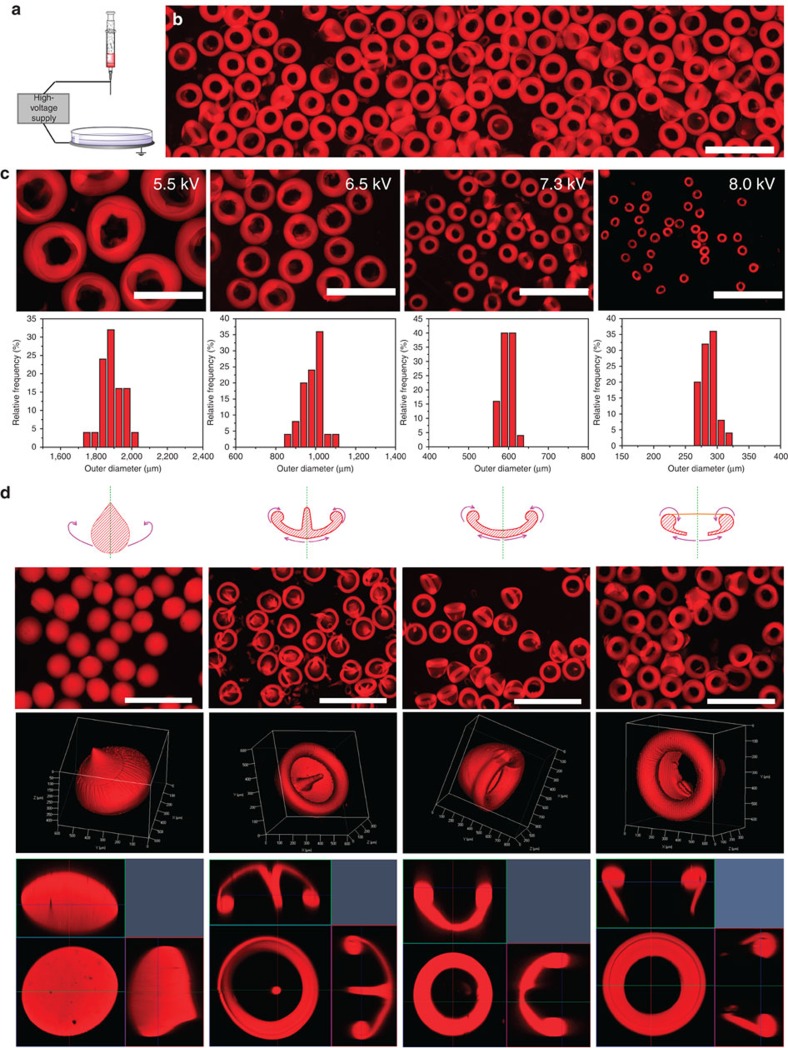
Mass production and size/shape control. (**a**) Schematic illustration of the electrospray setup. (**b**) Microscopic fluorescent images of the nanoclay hydrogel donut-microVRP fabricated by electrosparying. For better visualization, a fluorescent dye Rhodamine B was mixed in the nanoclay solution. (**c**) Size control of the nanoclay hydrogel donut-microVRP: microscopic images and corresponding size distribution plots. (**d**) Shape control of the nanoclay hydrogel microVRP by tuning the viscosity of the nanoclay solution and voltage: (from top to bottom) schematic illustrations, microscopic images, 3D confocal microscopic images and cross-sectional images. Scale bars, 2 mm (**b**); 1 mm (**c**,**d**).

The aforementioned simple dripping experiment proves the feasibility of freezing vortex rings at different stages to form various VRP. To reduce their dimensions and increase their production rate, we employ an industrially scalable electrospraying technique (Fig. 2a). Briefly, by adding a high voltage electric field between the nozzle and the collector, the liquid jet is charged and breaks up into much smaller droplets^34^. Interestingly, the fluid dynamic behaviour of smaller droplets impacting the crosslinking buffer remains similar to the ‘simple dripping' case. As a result, we easily fabricated nanoclay microVRP (for example, with the donut shape) at a very high rate (>15,000 VRP per min) (Fig. 2b). Both the micro-scale torus shape and the nanoscale porous network structure of these donut-microVRP are characterized and confirmed (Supplementary Fig. 1b–d).

Importantly, we discover that the size and shape of the electrosprayed VRP can be well controlled. By simply tuning the voltage, the outer diameter of the donut-microVRP can be controlled, from around 280 μm to over 1.5 mm (Fig. 2c). The microVRP dimensions are also controllable by tuning the working distance between the nozzle and the collecting buffer surface (Supplementary Fig. 4; Supplementary Note 3). Moreover, we can control the shapes of the microVRP by adjusting the viscosity of the nanoclay solution. Similar to the dripping method, we obtained, in addition to the donut-microVRP, three other types including teardrop-, jellyfish- and cap-shaped ones, all made with high uniformity and in mass numbers (Fig. 2d).

### Theoretical analyses and simulation

To better understand the control of size and shape of the nanoclay microVRP, we investigate theoretically how the size of the donut-microVRP or droplets is affected by the processing parameters in electrospraying such as the voltage. A classic scaling law which is valid for liquids with relatively low viscosities and electrical conductivities indicates the relationship between droplet size (*D*) and voltage (*V*)^34^:


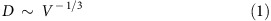


**Figure 3:**
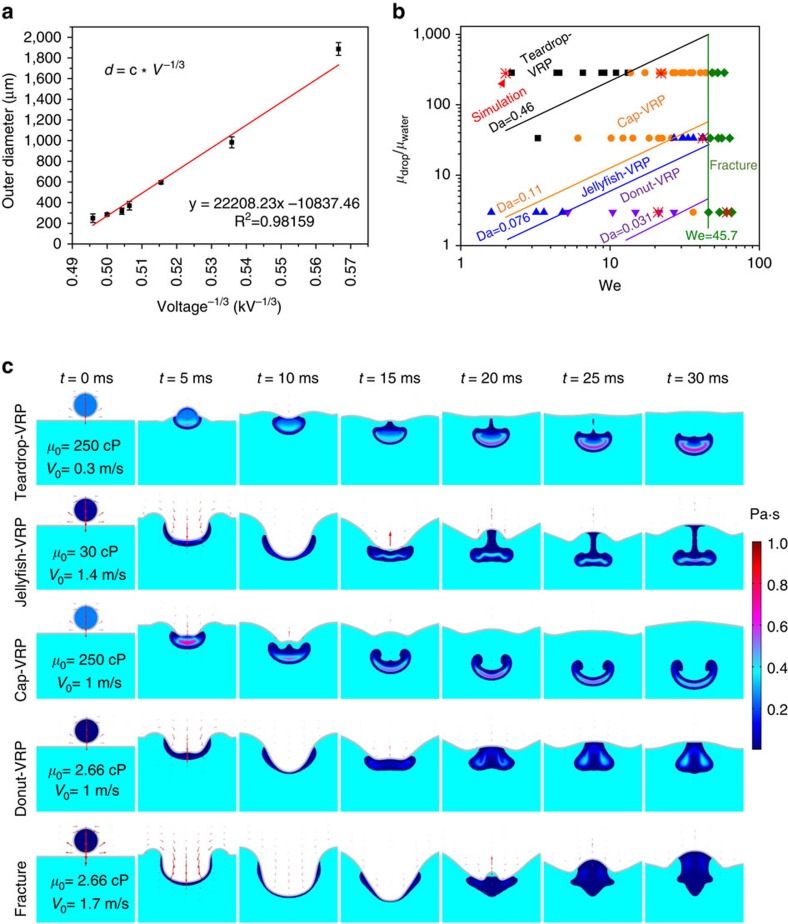
Mechanistic study and simulation. (**a**) MicroVRP sizes controlled by tuning voltage. Error bars in **a**, represent one standard deviation of the data away from the mean. (**b**) Experimental results and theoretical analysis of the VRP shapes for different parameters. (**c**) The axisymmetric simulation results of the vortex ring formation over time for five situations. The light blue is the crosslinking bath and the droplet above is the nanoclay solution where the colour represents the viscosity difference. The nanoclay solution is only plotted where there is at least 0.2 wt% nanoclay particles and the grey line at the air/solution interface represents where there is equal air–water volume fraction.

This scaling law suggests that the droplet size will decrease as the voltage increases. As shown in Fig. 3a, experimental data are plotted and fitted in this relationship assuming the droplet size is proportionally related to the VRP size. The fitting result indeed shows a linear relationship with a coefficient of determination *R*^2^≈0.9816.

Next, to understand the controllability of the VRP shape, we conducted a series of experiments and simulations to study how the properties of the drop before impact and after immersion dictated the development of vortex rings (Supplementary Figs 5–8; Supplementary Table 1; Supplementary Note 4). On impact, drop deformation is resisted by interfacial tension while, after immersion, VRP formation depends on the rate of mixing by invasion of vorticity into vorticity-free regions relative to the rate of reaction. Pre-impact ballistics is controlled by Weber (We) and Ohnesorge (Oh) numbers while post-impact vortical invasion and reaction is controlled by Reynolds (Re) and Damköhler (Da) numbers^6^,^8^ (Supplementary Note 6), defined as


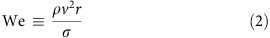



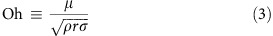



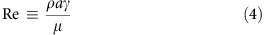



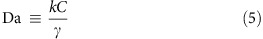


where *ρ* is the drop's density, *v* is the impact velocity, *r* is the radius, *μ* is the dynamic viscosity, *σ* is the surface tension, *k* is the reaction rate constant, *C* is the concentration of binding sites, and *aγ* is the rate of formation of interfacial area *a* between regions with and those without vorticity. Rate *γ* is estimated as a shear rate, from the simulation results. We and Oh numbers respectively characterize the strength of inertia and viscosity relative to surface tension of the impacting drops. Re and Da numbers respectively characterize rates of vorticity generation *aγ* relative to resistance to invasion of vorticity by viscosity *μ*/*ρ* and rate of crosslinking *kC* relative to shear rate *γ*.

The results from all experiments and simulations are summarized in Fig. 3b,c and Supplementary Information. According to our experimental and simulation results, with low-We, high-Oh and high-Da (that is, reaction dominates mass transport), the droplet is ‘frozen' at the early stage of vortex ring development and forms a teardrop VRP. While increasing We or decreasing Oh in the intermediate Da range (that is, reaction and mass transport are commensurate), cap- or jellyfish-VRP are obtained. Further increasing We created rotating vortices that punctures the cap's central part to produce a donut-VRP. When the We is large (or Oh low) and the Da sufficiently low (that is, mass transport dominates reaction), the particle cannot maintain its shape as a whole due to the reaction rate being much slower than the transport, the particle ends up fracturing (Supplementary Fig. 9). Normally, unconstrained liquid tori are very unstable and hence difficult to preserve. However, our method points out a simple approach of preserving any shape that a drop may form while impacting the free surface of a crosslinking agent. We, Oh, Re and Da are the lumped control parameters of solution properties and process. Our work suggests that, regardless what materials are used, an appropriate prescription of the four dimensionless numbers in principle will allow us to obtain a continuously deformed family of all the shapes spanning the developing process of a vortex ring. (Supplementary Note 5)

### MicroVRP formed with other materials and reactions

Our modelling and simulation suggests that microVRP can be fabricated from other materials as long as the deformation and ‘freezing' of vortex rings can both be controlled. To experimentally test this conclusion, we first chose two popular, crosslinkable biopolymers, alginate and chitosan. These two materials have been processed into hydrogel particles via electrospraying and they have been extensively used for drug and cell deliveries^35^. However, as in almost all previous studies, electrosprayed hydrogel microparticles have been spherical^36^,^37^. This is as expected according to our mechanistic studies due to the fact that their gelation reactions are relatively fast and that there is no time for the droplets to evolve into vortex rings. For instance, alginate droplet stops changing shape 20 ms after impacting a conventional crosslinking buffer (Supplementary Fig. 10). Decreasing the crosslinking buffer concentration may slow down the gelation, but the low concentration ion buffers cannot completely crosslink the hydrogel, resulting in fractures (Supplementary Tables 2 and 3; Supplementary Note 7).

**Figure 4:**
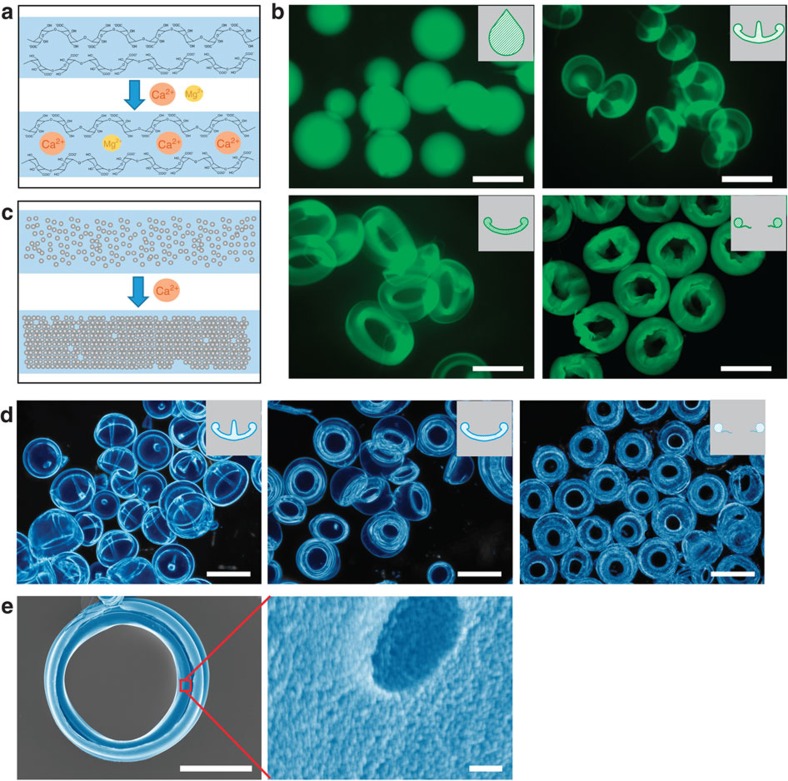
MicroVRP fabricated from different materials. (**a**) Scheme of the modified gelation process in the ion-competing crosslinking buffer for alginate. (**b**) Alginate hydrogel microVRP, alginate labelled with Alexa Fluor 488 dye. (**c**) Scheme of the silica nanocolloidal precipitation process. (**d**) Microscopic images of the nanosilica microVRP (false-coloured). (**e**) SEM images of a dried nanosilica donut-microVRP (false-colored). Scale bars, 500 μm (**b**,**d**); 60 μm (**e** left panel); 100 nm (**e**, right panel).

Here, to control the freezing event, we developed a novel ion-competing crosslinking solution containing a primary crosslinking agent (calcium ions for alginate or tripolyphosphate for chitosan) and a competing agent (magnesium ions or dihydrogen phosphate ions). Since the competing agent has a strong resemblance to the primary one but a much weaker crosslinking ability, when the droplet enters the ion-competing crosslinking solution, the competing agent competes with the primary one and hence slows down the reaction (Fig. 4a; Supplementary Fig. 11a). Indeed, using the ion-competing crosslinking solutions we successfully created microVRP from both alginate (Fig. 4b; Supplementary Fig. 12) and chitosan (Supplementary Fig. 11b).

More interestingly, we can fabricate microVRP from inorganic colloidal nanoparticles as well. Silica nanocolloidal solution (ca. 50 nm in diameter) is initially stabilized in sodium chloride buffer. When the ionic strength is changed, the electrostatic homeostasis among the silica nanoparticles is broken, causing the nanoparticles to aggregate and precipitate (Fig. 4c). This precipitation event is akin to the gelation event. As a result, by electrospraying the silica nanocolloidal solution into an ionic buffer (see Methods for details), we also achieved mass production of uniform nanosilica jellyfish-, cap- and donut-microVRP (Fig. 4d). These microVRP have excellent stability and rigidity; they remain almost intact after air-drying (Fig. 4e). Further energy dispersive spectrometer (EDS) element mapping reveals the uniform distribution of Si, O, Ca and Cl (Supplementary Fig. 13), confirming the relatively homogeneous precipitation.

### Hydorgel donut-microVRP for bioencapsulation

The fact that non-spherical, microVRP can be produced from a wide range of materials makes them not only fundamentally intriguing but also practically useful for real-world applications. For example, the donut-microVRP have several advantages over the spherical particles such as a larger surface area per volume, a shorter diffusion pathway within and sometimes a better deformability. These unique advantages combined with the tunable chemical composition make the donut-microVRP an interesting class of material for bioencapsulation applications.

**Figure 5:**
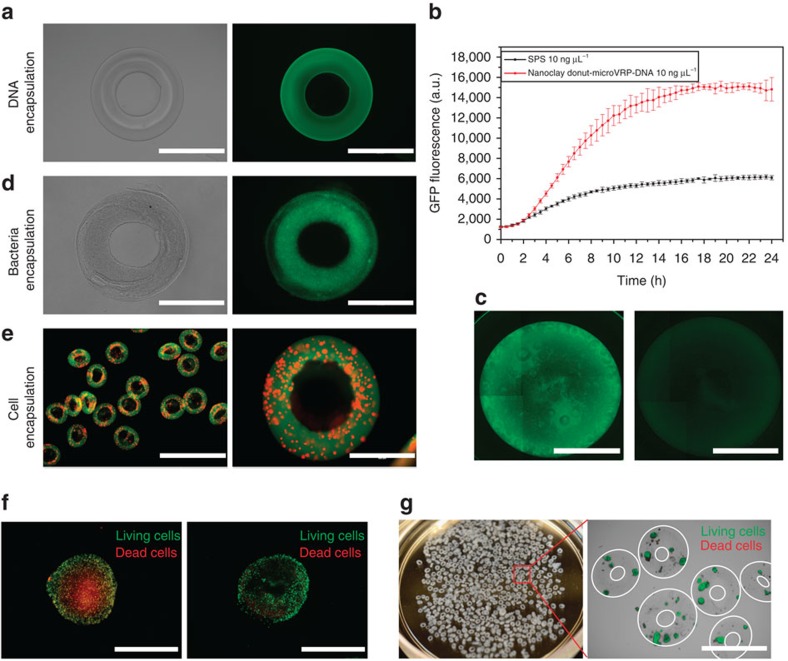
Bioencapsulation applications using donut-microVRP. (**a**) A nanoclay hydrogel donut-microVRP encapsulating plasmid DNA: bright-field image and fluorescent image (stained with GelGreen). (**b**) Kinetic curve of the GFP fluorescence from cell-free protein production. Error bars in **b** represent one s.d. of the data away from the mean. (**c**) Fluorescent images of the reaction wells of cell-free protein production with (left) or without (right) nanoclay donut-microVRP. (**d**) A nanoclay hydrogel donut-microVRP encapsulating bacteria: bright-field image and fluorescent image (GFP-expressing *E. coli*). (**e**) Alginate hydrogel donut-microVRP encapsulating MDA-MB-231 cells that express Tomato red. (**f**) Cell viability test of the MDA-MB-231 cells encapsulated in an alginate spherical particle and an alginate donut-VRP, 4 days after encapsulation. The cells were stained with calcium-AM (green, live) and ethidium homodimer (red, dead). (**g**) Rat pancreatic islets encapsulated in the alginate donut-VRP. Scale bars, 400 μm (**a**, **d**, **e** right panel); 3 mm (**c**); 2 mm (**e** left panel, **f**, **g**).

As a first proof-of-concept, we employed the nanoclay hydrogel donut-microVRP to encapsulate DNA molecules (Fig. 5a) for improved cell-free protein production. Our previous work shows that the bulk nanoclay hydrogel protects DNA from DNase and hence enhance the cell-free protein production yield^30^. Compared with bulk nanoclay hydrogel, the donut-microVRP have a much higher surface area for mass transfer and are therefore ideal for protein production applications. Here, using a green fluorescent protein (GFP) as a reporter, the nanoclay donut-microVRP-DNA group produces more than three times of GFP than the conventional solution-phase system (Fig. 5b,c).

Next, we demonstrate efficient encapsulation of cells (both prokaryotes and eukaryotes) in hydrogel donut-microVRP. GFP-expressing *Escherichia coli* cells were encapsulated in the nanoclay hydrogel donut-microVRP (Fig. 5d) and interestingly, the encapsulation did not affect the proliferation of the *E. coli* (Supplementary Fig. 14) and bacterial donut-microVRP were obtained. Additionally, we encapsulated mammalian cells or cell aggregates in alginate hydrogel donut-VRP (Fig. 5e). Alginate hydrogel has been widely used for cell encapsulation for various applications such as type 1 diabetes treatment^38^,^39^,^40^. The hydrogel provides an immuno-isolated environment for cells while allowing nutrients, oxygen and therapeutics to freely diffuse in and out. For decades, almost all previous works have used spherical alginate particles, typically with a diameter of 500–600 μm. However, recent studies have shown that it is actually more desirable to use larger size particles (about 1.5 mm or larger in diameter) for cell delivery because these larger particles generate less foreign body responses upon implantation^41^. Unfortunately, for the large, spherical particles, mass transfer becomes a real problem (for example, the cells in the centre of the spheres can experience the lack of nutrients and oxygen). The non-spherical, donut-VRP, on the other hand, are promising candidates to replace the spherical particles due to their larger surface area and shorter diffusion pathway within (that is, decoupling of the overall dimension and the diffusion distance). To prove the concept, we encapsulated MDA-MB-231 cells, a human breast cancer cell line, in both alginate hydrogel spherical particles and donut-VRP. After four days of culture, the cells in the centre of the spherical particles started to die, while the cells in the donut-VRP remained mostly alive (Supplementary Fig. 15; Fig. 5f). Encouraged by this result, we further demonstrate the feasibility of encapsulating rat pancreatic islets in the alginate hydrogel donut-VRP. The encapsulated islets again showed promising viability after 2 days *in vitro* culture (Fig. 5g).

### Composite and assembly of donut-microVRP

**Figure 6:**
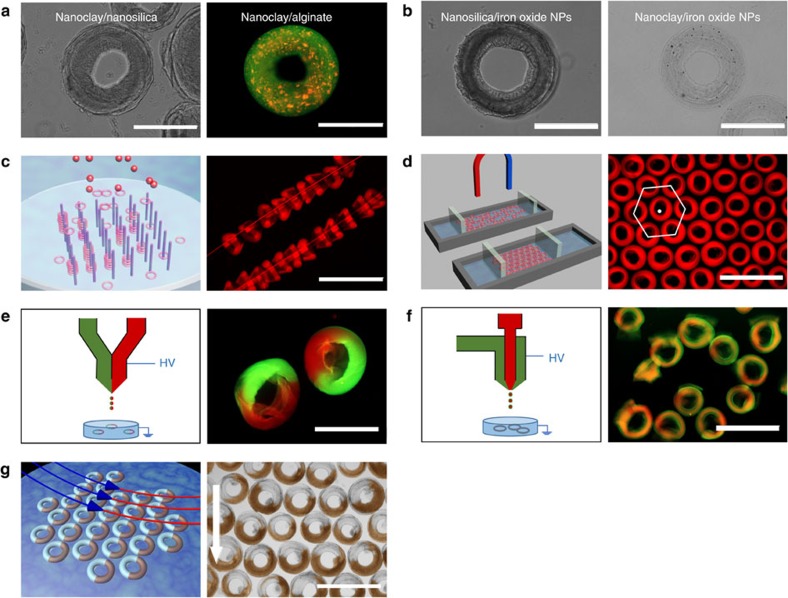
Composite and assembly of donut-microVRP. (**a**) Donut-microVRP made from composite materials: nanoclay/nanosilica, nanoclay/alginate. (**b**) Magnetic donut-microVRP: nanosilica/Fe_3_O_4_ nanoparticles and nanoclay/Fe_3_O_4_ nanoparticles. (**c**) Schematic illustration of the assembly process and a microscopic image of the 1D structure made from nanoclay hydrogel donut-microVRP. (**d**) Schematic illustration of the assembly process and a microscopic image of the 2D structure made from magnetic nanoclay hydrogel donut-microVRP. (**e**) Schematic illustration of the nozzle design and a microscopic image of the Janus donut-microVRP. (red: alginate labelled with Alexa Fluor 594 dye; green: alginate labelled with Alexa Fluor 488 dye) (**f**) Schematic illustration of the nozzle design and a microscopic image of the core–shell donut-microVRP. (**g**) Directional assembly of the Janus magnetic nanosilica donut-microVRP; the arrow indicating the direction of the magnetic field. Scale bars, 400 μm (**a**,**b**); 2 mm (**c**,**d**,**f**); 1 mm (**e**,**g**).

Composite materials often possess unique properties due to synergistic combination of the characteristics of the components. They have been widely used in applications such as catalysis, sensing, actuation, computation and communications^42^. To further enhance and diversify the properties and functionalities of the donut-microVRP, we applied the approach of ‘freezing' to make VRP with even more complicated compositions and structures. First, we demonstrate two different kinds of donut-microVRP made from composite materials: nanoclay/nanosilica and nanoclay/alginate (Fig. 6a). Addition of nanosilica or alginate improves the mechanical strength of the microVRP while maintaining the special characteristics of the nanoclay hydrogel such as enhancement of cell-free protein production. This combination can potentially make the nanoclay/nanosilica donut-microVRP applicable for packing a packed bed reactor for continuous cell-free protein production^43^,^44^. In addition, we also doped other materials into the current donut-microVRP to introduce new properties. For examples, Fe_3_O_4_ nanoparticles were doped in the nanosilica or nanoclay matrix to prepare magnetic donut-microVRP (Fig. 6b), which can be manipulated using an external magnetic field and potentially used as building blocks for novel stimuli-response materials^45^.

Next, by taking advantage of the unique donut-shape, we directionally assembled the donut-microVRP in either one or two dimensions into supra-structures. For one-dimensional (1D) assembly, we made use of the holes of the donut-microVRP. As shown in the scheme in Fig. 6c, several Nylon short strings were fixed perpendicular to the air-solution interface. When the donut-microVRP formed, they were threaded into Nylon strings and captured *in situ* into 1D assemblies (Fig. 6c). For two-dimensional assembly, we assembled the magnetic donut-microVRP into a close-packed monolayer with hexagonal packing symmetry by a combination of magnetic field and a monolayer assembly strategy similar to that used in making Langmuir–Blodgett films (Fig. 6d).

Moreover, by engineering the nozzle used in electrospraying, we fabricated donut-microVRP with compartmentalized structures. As shown in Fig. 6e, the Janus alginate hydrogel donut-microVRP are produced by using a side-by-side nozzle. Core–shell alginate hydrogel donut-microVRP are also successfully prepared by using a core–shell nozzle (Fig. 6f). These bi-compartmental donut-microVRP may find a wide range of applications in biomedical applications, such as co-culture and co-delivery of different types of cells. Additionally, we show another example of magnetic field-assisted directional assembly of Janus donut-microVRP. Janus donut-microVRP with half nanosilica and half nanosilica/Fe_3_O_4_ nanoparticles are prepared using a side-by-side nozzle. They are aligned under an external magnetic field. Due to their asymmetric magnetic property, all the donut-microVRP are oriented in the same direction with the magnetic field (Fig. 6g).

## Discussion

Compared with spherical particles, non-spherical particles have received much less attention in materials community. However, non-spherical particles such as the donut-shaped ones are often advantageous due to their larger surface areas and shorter diffusion distances. Therefore, mass production of non-spherical particles with controlled dimensions and geometries from different materials will have important implications in fields ranging from catalysis to therapeutic delivery. Here, we report, for the first time, the creation of a new class of non-spherical particles, vortex ring-derived particles. By combining the ubiquitous vortex ring phenomenon with rationally designed freezing events, we develop a universal, robust and scalable method to produce uniform VRP from a variety of materials. Mechanistic studies reveal two critical timescales that determined the VRP shape: the timescale of the vortex ring development and the timescale of the gelation or precipitation process. In order for the VRP to form, the solution properties and process parameters must be well tuned such that the fast evolving vortex rings can be frozen at different stages. Among the various VRP, the donut-VRP are of particular interest and their diverse applications in bioencapsulation and cell-free protein production are demonstrated. Further engineering of donut-VRP to include compartmentalization and directional assembling are all achieved, creating opportunities to design and engineer VRP-based novel materials that have not been attainable using conventional methods.

## Methods

### Materials

All chemicals were used as received. Nanoclay powders (Laponite XLG) were received from Southern Clay Products. Sodium alginate (PRONOVA UP VLVG) was purchased from FMC Corporation. Colloidal silica (LUDOX TM HS-40), chitosan (low molecular weight), Sodium tripolyphosphate, CaCl_2_, MgCl_2_ and acetic acid were purchased from Sigma-Aldrich. NaH_2_PO_4_ was purchased from J.T. Baker.

### Preparation of VRP and microVRP

For nanoclay hydrogel VRP, typically, a droplet of 2% (w/v) nanoclay solution was extruded from a syringe needle. The droplet fell from a certain height into a 100 mM CaCl_2_ buffer. The viscosity of the nanoclay solution was tuned by changing the CaCl_2_ concentration in the nanoclay solution. For nanoclay hydrogel donut-microVRP, a 2% (w/v) nanoclay solution was electrosprayed into the 100 mM CaCl_2_ solution at a voltage of 7.3 kV with a pumping rate of 0.06 ml min^−1^. The working distance between the nozzle and collecting buffer surface was fixed at 4 cm. For typical alginate hydrogel donut-microVRP, 2% (w/v) sodium alginate solution was electrosprayed into an aqueous buffer of 5 mM CaCl_2_ and 95 mM MgCl_2_ at 4.7 kV with a pumping rate of 0.06 ml min^−1^. For typical chitosan hydrogel donut-microVRP, 0.5% (w/v) chitosan/acetic acid solution was electrosprayed into acetic acid buffer containing 13.6 mM sodium tripolyphosphate and 50 mM KH_2_PO_4_. Voltage was fixed at 4.8 kV and the pumping rate was 0.3 ml min^−1^. For nanosilica donut-microVRP, silica nanocolloidal solution was directly electrosprayed into a 100 mM CaCl_2_ aqueous buffer at 4.6 kV with a pumping rate of 0.06 ml min^−1^. Other microVRP shapes were obtained by tuning the nanoclay/alginate/chitosan/silica nanocolloidal solution viscosity or electrospraying voltage.

### Characterizations

The samples were characterized by different analytical techniques. Scanning electron microscopy (SEM) and EDS element mapping were performed by using a field emission scanning electron micro-analyzer (LEO 1550). Optical and fluorescent microscopic images were observed by a digital inverted microscope (EVOS fl). Confocal laser scanning microscopy (CLSM) images were taken by confocal laser scanning microscope (ZEISS LSM710). High-speed camera images were captured by a high-speed camera (RedLake HG-XL, Integrated Design Tools).

### Cell/bacteria encapsulation

For cell/bacteria encapsulation, MDA-MB-231 cells, isolated rat pancreatic islets or *E. coli* were collected by centrifugation, after removing the supernatant, cells or cell aggregates were re-suspended into a 2% sodium alginate solution. This cell/alginate solution was electrosprayed into the 95 mM MgCl_2_/5 mM CaCl_2_ buffer. Then, the donut-microVRP were collected and washed by PBS for three times to remove excess crosslinking buffer and transferred into corresponding culture medium.

### Cell-free production of model protein

To produce green fluorescent protein (GFP), 1.4 μg of expression plasmid (PIVEX 2.4 containing GFP (5PRIME)) was added to a solution containing 5 mg of nanoclay hydrogel donut-microVRP, 37.8 μl of cell-free lysate, and 15 μl of nuclease free water. Samples were then placed in a BioTek fluorescent plate reader and measured every 30 min with an excitation of 488 nm and an emission of 509 nm. In order to determine the effect of nanoclay hydrogel donut-microVRP on protein expression, samples were done in triplicate and side-by-side with control samples. All samples were then imaged at the 24-h time point.

### Data availability

The data that support the findings of this study are available from the corresponding author upon request.

## Additional information

**How to cite this article:** An, D. *et al*. Mass production of shaped particles through vortex ring freezing. *Nat. Commun.* 7:12401 doi: 10.1038/ncomms12401 (2016).

## Supplementary Material

Supplementary Information Supplementary Figures 1-15, Supplementary Tables 1-3, Supplementary Notes 1-7, Supplementary Methods and Supplementary References
