# Supplementary material for: Mass production of shaped particles through vortex ring freezing
Source: Nat Commun. 2016 Aug 4;7:12401. doi: 10.1038/ncomms12401 (PMC4976289; doi:10.1038/ncomms12401)
Supplement: Supplementary Information — Supplementary Figures 1-15, Supplementary Tables 1-3, Supplementary Notes 1-7, Supplementary Methods and Supplementary References [file ncomms12401-s1.pdf]

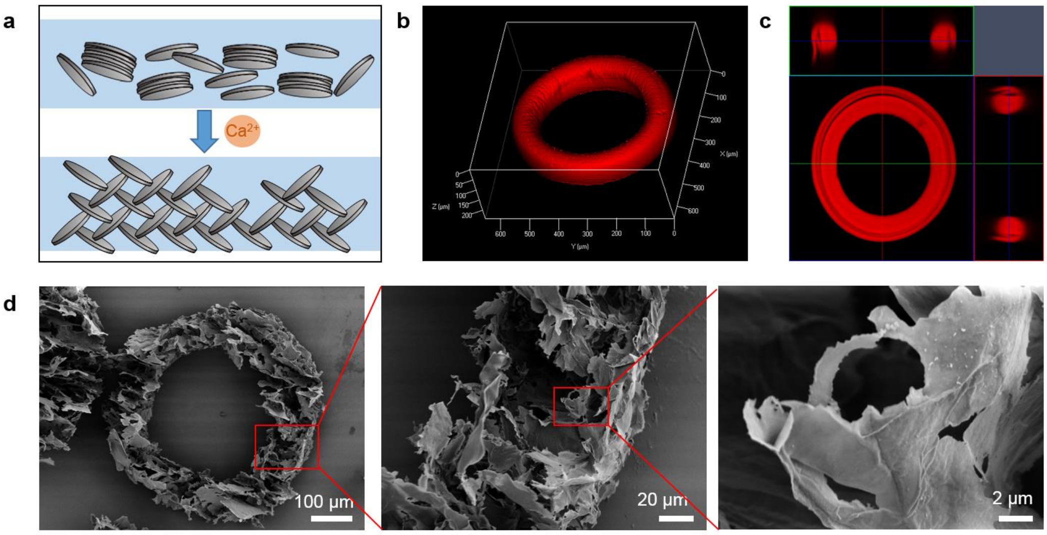

Supplementary Figure 1. Characterizations of nanoclay hydrogel donut-microVRP. (**a**) Schematic illustration of the gelation process of nanoclay hydrogel. (**b**, **c**) Confocal microscope images of a nanoclay hydrogel donut-microVRP: 3-D reconstruction (**b**) and a cross-sectional image(**c**). (**d**) SEM images of a freeze-dried nanoclay hydrogel donut-microVRP at different magnifications. Scale bars in (**d)** (from left to right): 100 μm, 20 μm, 2 μm.

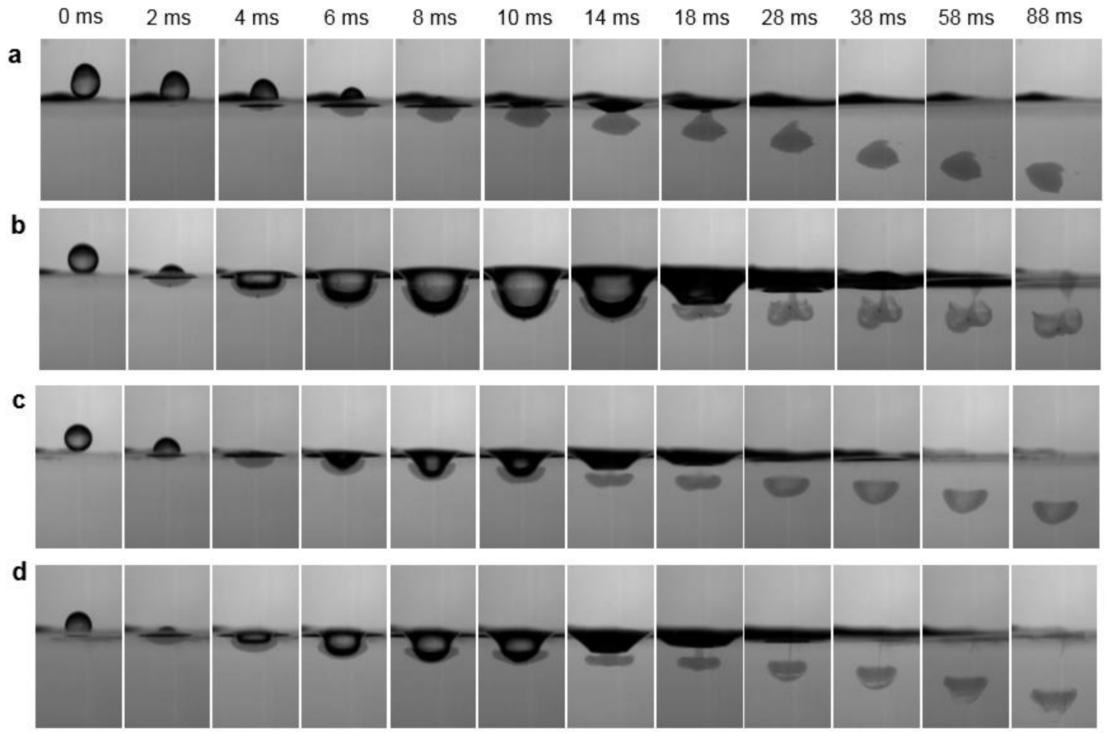

Supplementary Figure 2. High speed camera images of the formation processes of the nanoclay hydrogel VRP. (**a**) teardrop- (**b**) jellyfish- (**c**) cap- and (**d**) donut-VRP.

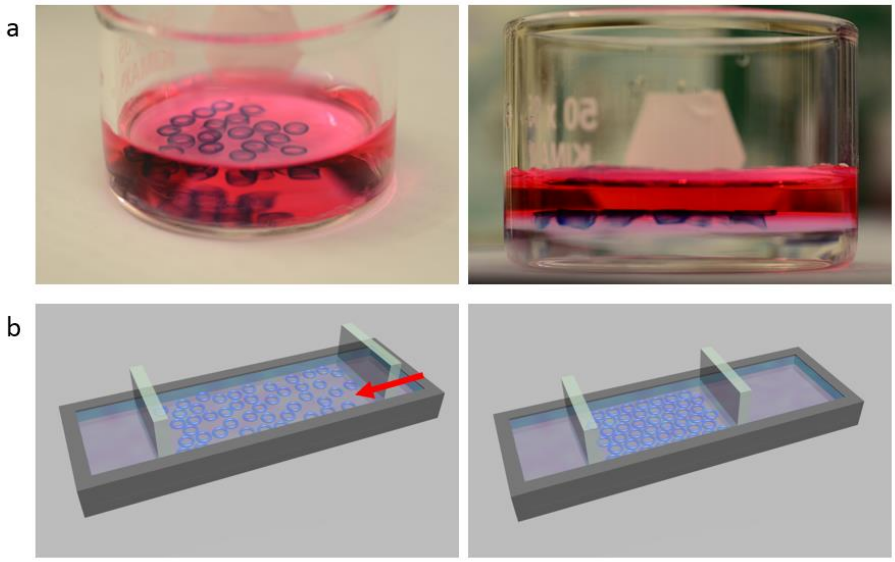

Supplementary Figure 3. Monolayer assembly of donut-VRP. (**a**) Digital images showing the nanoclay hydrogel donut-VRP at the interface between DMEM buffer (red) and Histopaque ® (clear). (**b**) Schematic illustration of the assembly strategy.

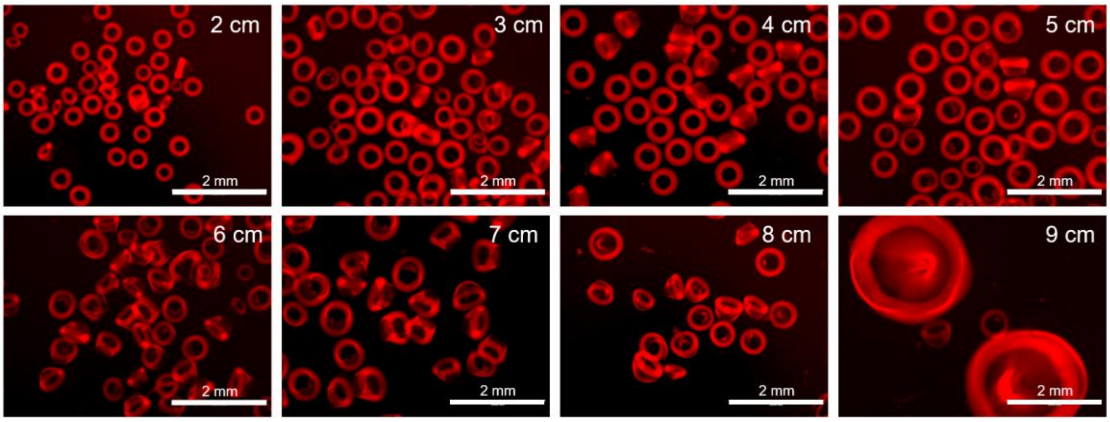

Supplementary Figure 4. Microscopic images of the nanoclay micro-VRP produced at different electrospray working distances. Scale bars: 2 mm.

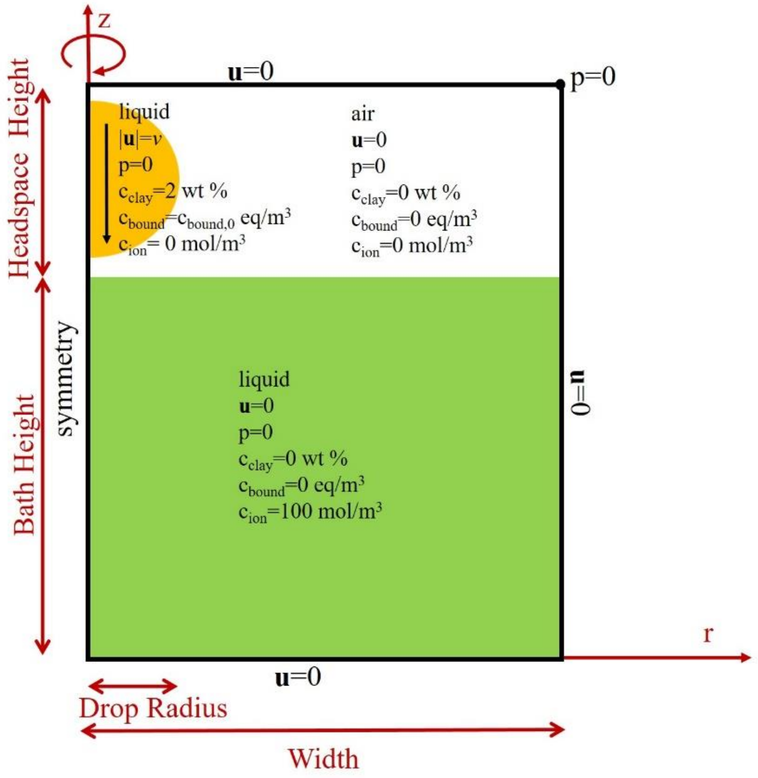

Supplementary Figure 5. Schematic of initial and boundary conditions for axisymmetric model. The fluids are water and air. The yellow is the bound nanoclay in water, white is air, and green is the ion water bath.

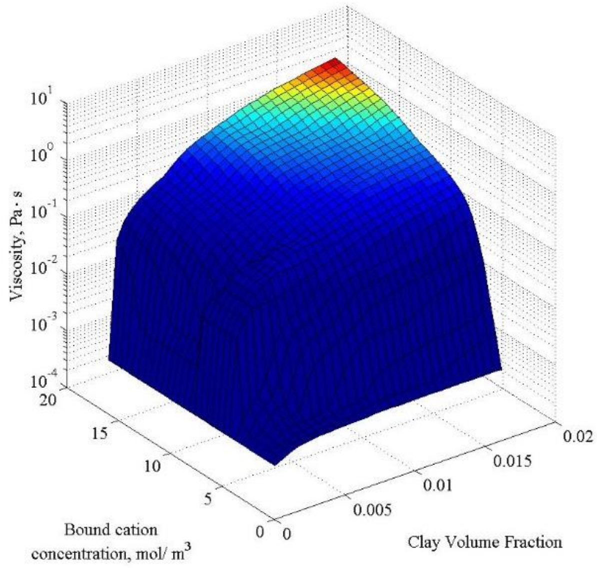

Supplementary Figure 6. Viscosity as a function of nanoclay volume fraction and bound cation concentration.

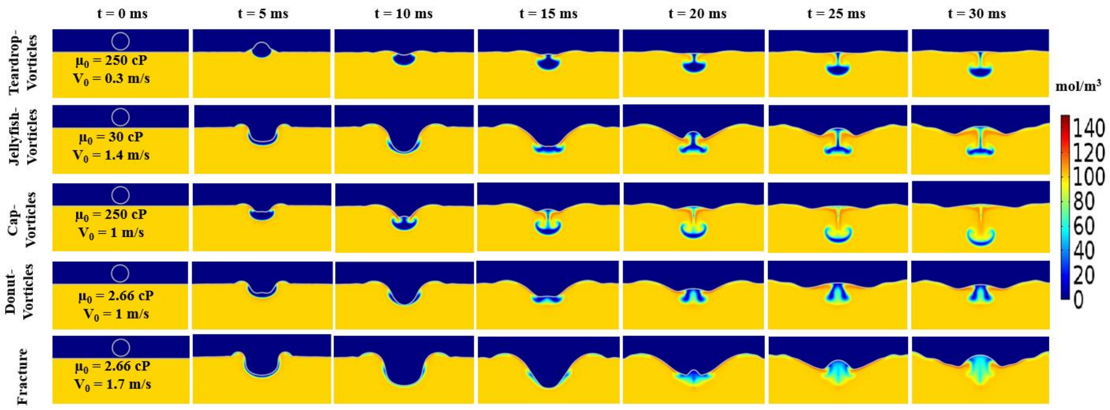

Supplementary Figure 7. The ion concentration with time for various initial conditions shown in Figure 3.

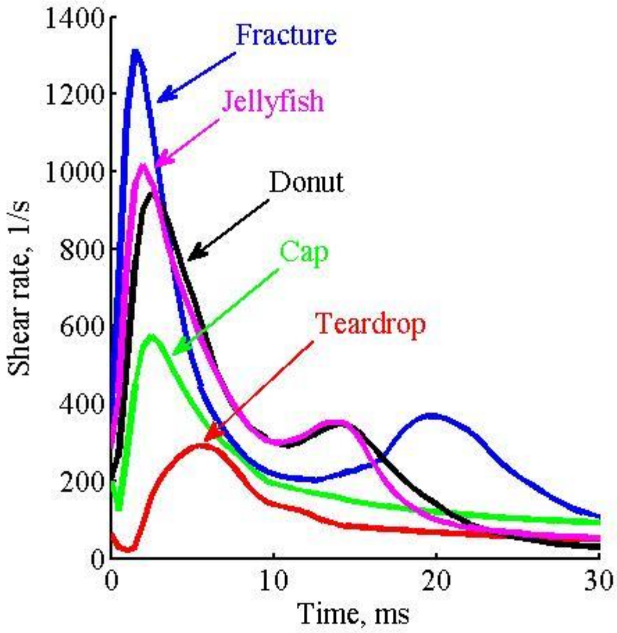

Supplementary Figure 8. The weighted average shear rate for each structure type from Figure 3.

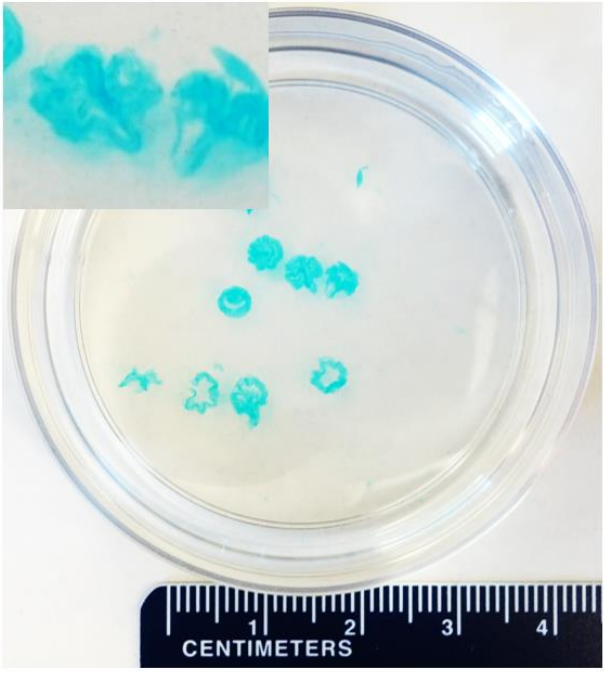

Supplementary Figure 9. Nanoclay hydrogel fractures.

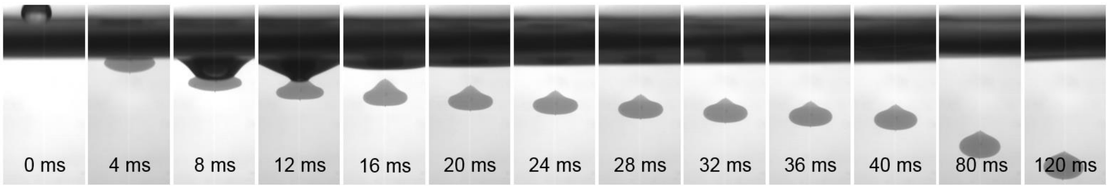

Supplementary Figure 10. High speed camera images of an alginate solution impacting the conventional crosslinking buffer (100 mM $CaCl_2$ solution).

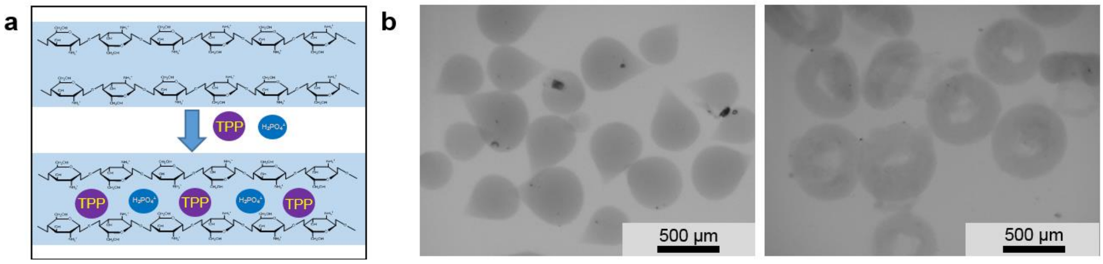

Supplementary Figure 11. (**a**) Scheme of the modified gelation process for chitosan with the ion-competing crosslinking buffer. (**b**) Chitosan hydrogel microVRP.

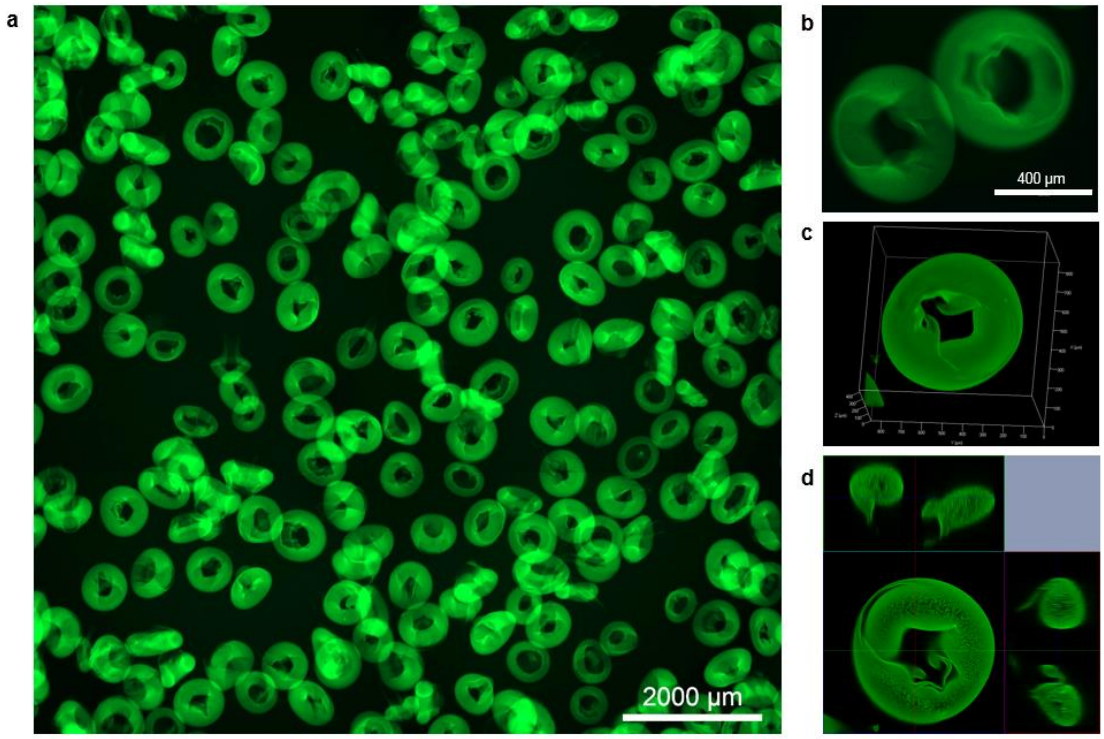

Supplementary Figure 12. Fluorescent microscope images of alginate hydrogel donut-microVRP. (**a**,**b**) Donut-microVRP made of Alexa Fluor® 488 labeled alginate hydrogel at different magnifications. (**c**) Reconstructed 3-D image of an alginate hydrogel donut-microVRP. (**d**) Cross-sectional images of an alginate hydrogel donut-microVRP.

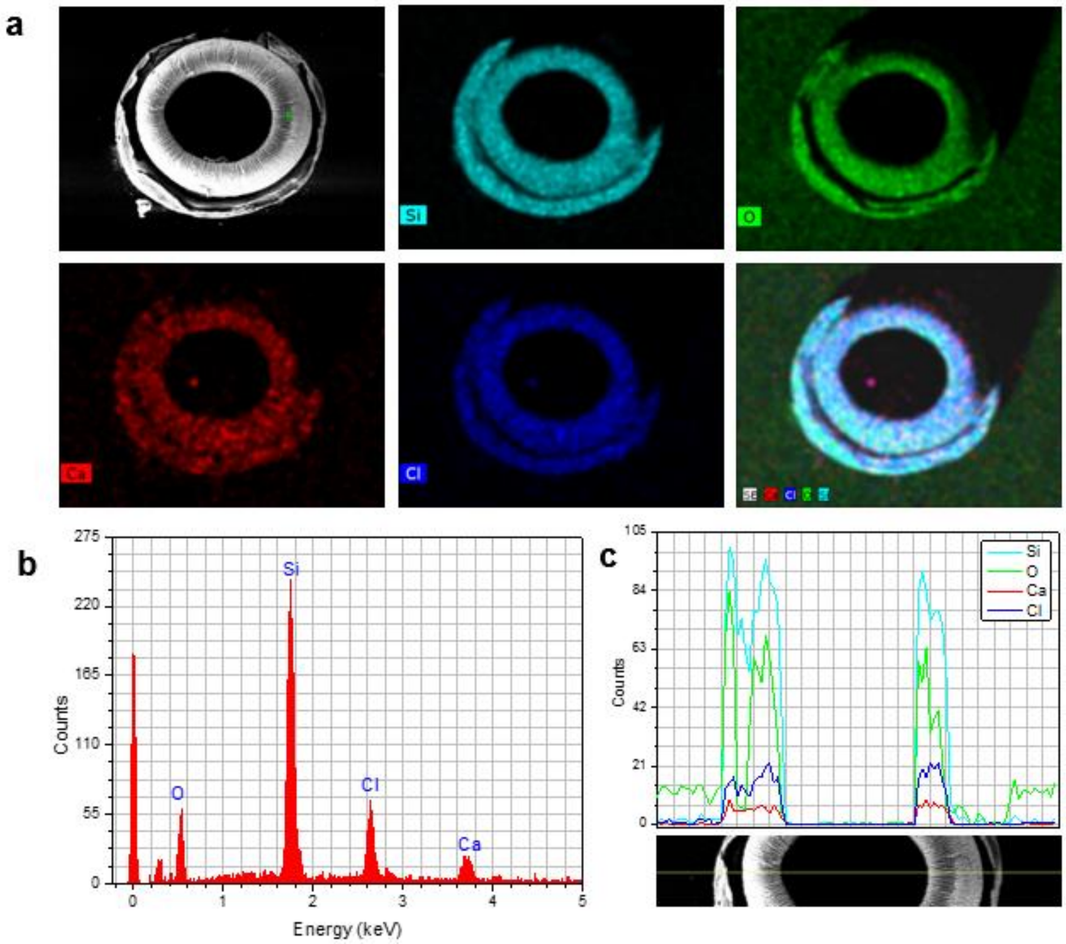

Supplementary Figure 13. (**a**) EDS mapping of a nanosilica donut-microVRP showing element distribution of Si, O, Ca and Cl. (**b**) Energy dispersive x-ray (EDS) spectrum of the nanosilica donut-microVRP. (**c**) Line scan analysis of the nanosilica donut-microVRP.

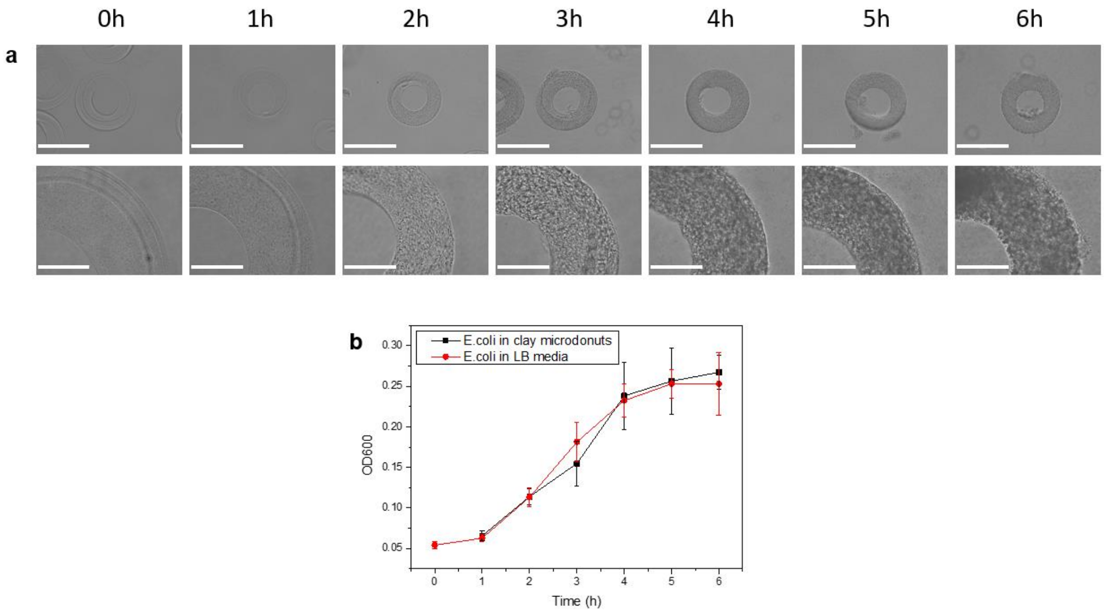

Supplementary Figure 14. Bacteria proliferation test within the nanoclay hydrogel donut-microVRP. (**a**) Microscopic images of the nanoclay hydrogel donut-microVRP encapsulating *E.coli*. (**b**) Growth curve of the *E.coli* encapsulated in the nanoclay hydrogel donut-microVRP. Scale bars, 400 μm (upper panel); 100 μm (lower panel)

Certain amount of *E.Coli* were uniformly dispersed in nanoclay solution at a predetermined concentration. Then the solution was electrosprayed into Lysogeny broth (LB) medium to make nanoclay donut-microVRP. The obtained donut-microVRP were transferred into a 96 well plate and cultured at 37°C. Same amount of *E.coli* were cultured in LB medium without encapsulation. The bacteria amount was monitored by both microscope and measuring OD600.

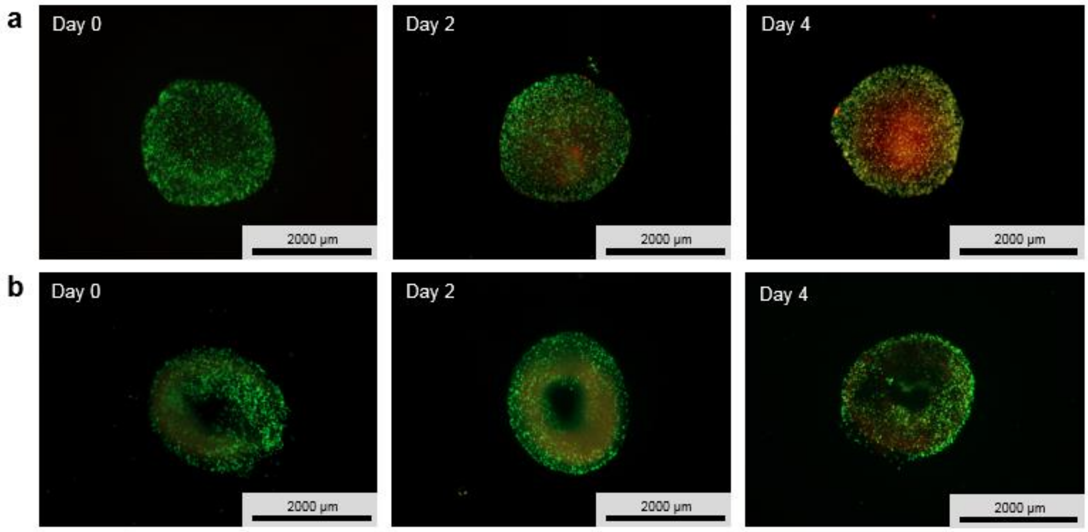

Supplementary Figure 15. Cell viability test of MDA-MB-231 cells encapsulated in (**a**) a spherical alginate particle and (**b**) an alginate donut-VRP. Cells were stained with calcium-AM (green, live) and ethidium homodimer (red, dead).

**Shape**	**Average Da**	**Standard deviation Da**
**Teardrop**	0.046	0.013
**Cap**	0.024	0.0040
**Jellyfish**	0.02	0.0043
**Donut**	0.018	0.0033
**Fracture**	0.015	0.0029

Supplementary Table 1: Average and standard deviation Dahmköhler numbers for each shape based on a reaction time scale of 16.2 (1/s).

Concentration (mM)	$CaCl_2$(50)	$CaCl_2$(20)	$CaCl_2$(10)	$CaCl_2$(5)
2% VLVG<br>4.7 kV<br>0.6 mL/min	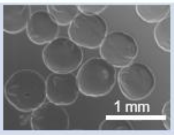	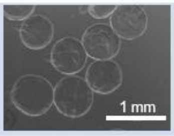	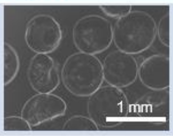	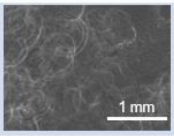
	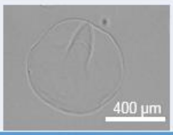	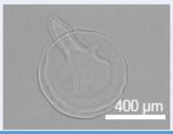	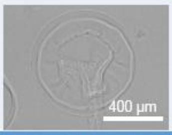	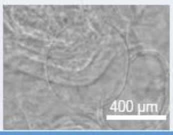
**Concentration (mM)**	**$CaCl_2$(50)/$MgCl_2$(100)**	**$CaCl_2$(20)/$MgCl_2$(100)**	**$CaCl_2$(10)/$MgCl_2$(100)**	**$CaCl_2$(5)/$MgCl_2$(100)**
	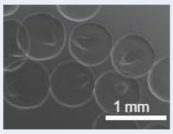	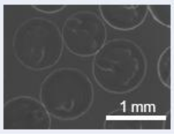	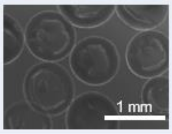	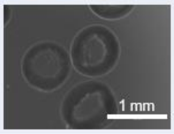
	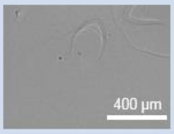	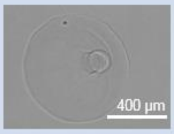	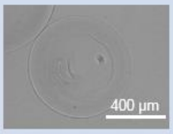	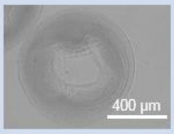

Supplementary Table 2. Different shapes of the electrosprayed alginate hydrogel microparticles using different crosslinking buffers.

Concentration (mM)	tripolyphosphate(54)	tripolyphosphate(27)	tripolyphosphate(13.6)	tripolyphosphate(6.8)
0.5% Chitosan/acetic acid<br>4.8 kV<br>0.3 mL/min	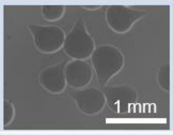	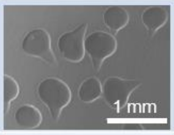	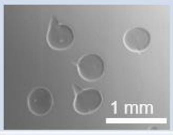	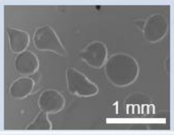
	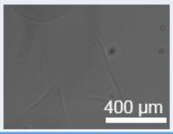	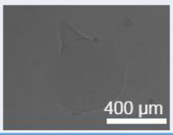	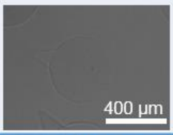	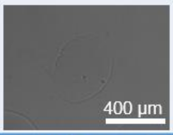
**Concentration (mM)**	**TPP(54)/KH_2PO_4(50)**	**TPP(27)/KH_2PO_4(50)**	**TPP(13.6)/KH_2PO_4(50)**	**TPP(6.8)/KH_2PO_4(50)**
	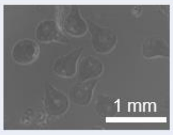	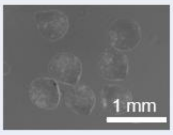	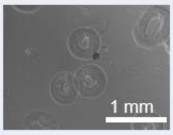	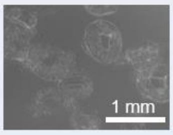
	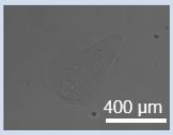	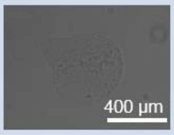	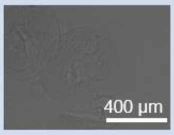	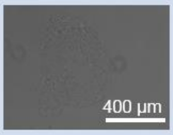

Supplementary Table 3. Different shapes of the electrosprayed chitosan hydrogel microparticles using different crosslinking buffers.

Supplementary Note 1

Discussion on the concept of "freezing"

In materials science, "freezing" typically refers to a phase transition in which a liquid solidifies or turns into a solid when its temperature is decreased below its freezing point. In this work, we use the term of "freezing" in a more general sense; that is to "fix a shape". More specifically, we use the term to describe a process that fixes the unstable or flowable liquid vortex rings into stable or non-flowable hydrogel or solid microparticles of a defined shape. This process could be a chemical reaction, physical gelation (crosslinking) or precipitation.

Supplementary Note 2

Assisted-assembly of nanoclay hydrogel donut-VRP into a close-packed monolayer

Due to the unique anisotropic symmetry of the nanoclay hydrogel donut-VRP, when floating on the liquid surface, all the nanoclay hydrogel donut-VRP preserved the same orientation. By taking advantage of this interesting property, we designed a "Langmuir-Blodgett deposition - like" assembly strategy to assembly the nanoclay hydrogel donut-VRP into a close-packed monolayer. Firstly, we discover that the nanoclay hydrogel donut-VRP can float at the interface between Dulbecco's Modified Eagle Medium (DMEM) and Histopaque, a commonly used cell separation system based on the density difference (Supplementary Fig. 3a). Then, this floating system was introduced into a "Langmuir-Blodgett deposition - like" device (Supplementary Fig. 3b). Basically, two barriers were used to push the floating donut-VRP closer into a close-packed monolayer. Other nanoclay hydrogel VRP were assembled using the similar strategy.

Supplementary Note 3

Effect of the electrospray working distance on the microVRP size/shape

We have studied the effect of working distance (i.e. the distance between the electrospray tip and the surface of the collecting solution) on the microVRP size and shape (Supplementary Fig. 4). The voltage was set at 7.5 kV and the same nanoclay solution was electrosprayed at different working distances ranging from 2 cm to 9 cm. In general, we observed relatively uniform donut-microVRP with increasing outer diameter when the working distance varied from 2 cm to 5 cm. This is consistent with the trend we observed when we fixed the working distance but decreased the voltage (Fig. 2c). This was expected since increasing working distance has a similar effect on the electric field strength to decreasing the voltage. However, when the working distance was higher than 6 cm, the electrospray jet became unstable and resulted in non-uniform microVRP with various sizes and shapes. Further increase of the working distance to 9 cm, the electrospray jet became so unstable that no valid experiment could be performed. From these experiments, we conclude that it may be difficult to control the shape of microVRP by tuning the working distance alone. A better way to control the shape is through the adjustment of the viscosity of the solution as shown in Fig. 2d.

Supplementary Note 4

Simulations

Model Overview, Assumptions and Geometry

The simulation uses an axisymmetric domain that is enclosed on all sides. A droplet with nanoclay bound at different ion concentrations (which leads to a different initial viscosities) starts slightly above the ion-bath. The droplet has an initial downward velocity while the bath is static. Supplementary Fig. 5 shows the axisymmetric model geometry and boundary and initial conditions of the simulation. The yellow is the bound nanoclay in water, white is air, and green is the ion water bath. The droplet radius was 1.5 mm for all simulations. The droplet height above the bath was 0.4 mm. The width of the domain was 12 mm while the height of the bath was assumed to be 8 mm with a headspace height of 4 mm.

The air and water interphase was tracked with the Cahn-Hilliard two fluid-phase field flow model[1, 2, 3]. The dispersed species, nanoclay, calcium, and nanoclay-calcium bound material, were modeled with a convection-diffusion mass transport model. The fluid velocity and pressure were modeled with an incompressible Newtonian fluid[4]. The dynamics of both fluid-phases and the dispersed species were fully coupled.

The surface tension between fluids was assumed to be (constant at 293 K) that of air/water mixture (0.0728 N/m) since there was only a 5% decrease in surface tension for a bound concentration of 4 eq m^{-3} when $CaCl_2$ was initially added. The nanoclay particles were assumed to be spherical and have a particle diameter, d_p, of 9.5 nm[5]. The reaction rate of bound nanoclay was iterated for until the structures in the simulation

were similar to those of the experiment. There was no assumed off reaction rate between the calcium and binding site. The dimensions were chosen based on the trade-off between computational time and boundary effects. Increasing domain sizes decreases the effect the wall has on the simulation but increases the computation time.

Governing Equations

Fluid Flow – Navier Stokes

The fluids' velocity and pressure are solved for with the Navier-Stokes equation (1) and assuming an incompressible fluid for the continuity equation (Eq. 2). The fluids are assumed to be air, *a*, and water, *l*, with dispersed phases in the liquid as discussed below,

$$\rho\frac{\partial \mathbf{u}}{\partial t} + \rho(\mathbf{u}\cdot\nabla)\mathbf{u} = \nabla\cdot\boldsymbol{\sigma}_u + \rho\mathbf{g} + \mathbf{F}_{ST} \quad (1)$$

$$\nabla\cdot\mathbf{u} = 0. \quad (2)$$

The two body forces exerted on both fluids are from gravity, **g**, and surface tension, $\mathbf{F}_{ST}$.

The stress tensor has two components, the pressure and viscous stress where **e**(**u**) is the strain tensor.

$$\boldsymbol{\sigma}_u = -p\mathbf{I} + 2\mu\mathbf{e}(\mathbf{u}) \quad (3)$$

$$\mathbf{e}(\mathbf{u}) = 0.5\left[\nabla\mathbf{u} + (\nabla\mathbf{u})^T\right] \quad (4)$$

The fluids properties are volume averaged based on the volume fraction of air, $V_{f,a}$.

$$\rho = \rho_l + (\rho_a - \rho_l)V_{f,a} \quad (5)$$

$$\mu = \mu_l + (\mu_a - \mu_l)V_{f,a} \quad (6)$$

Non-Newtonian Viscosity-Cross Model

Based on experimental data, the viscosity (Supplementary Fig. 6) was modeled as a function of the bound concentration of nanoclay and volume fraction of clay particles (Eq. 7).

$$\mu_p = \mu_p\left(\upsilon_{clay}, c_{bound}\right) \tag{7}$$

The viscosity (Eq. 8) of the liquid is taken as the sum of the two fluids and the density (Eq. 9) is based on the volume fraction (Eq. 10) of nanoclay particles in the liquid. Thus, the nanoclay solution is not neutrally buoyant and will sink as the density of nanoclay was 2530 kg m^{-3} [5].

$$\mu_l = \mu_s + \mu_p \tag{8}$$

$$\rho_l = \left(1 - \upsilon_{clay}\right)\rho_s + \upsilon_{clay}\rho_{clay} \tag{9}$$

$$\upsilon_{clay} = \frac{\frac{c_{clay}}{\rho_{clay}}}{\frac{c_{clay}}{\rho_{clay}} + \frac{1-c_{clay}}{\rho_w}} \tag{10}$$

Phase Tracking – Cahn-Hilliard

The governing equation for tracking the fluid phases (air and water) and their interface is the Cahn-Hilliard equation[8] (Eq. 11) with ψ as the chemical potential (Eq. 12). The variable, ϕ, is the phase field variable and is directly related to the volume fractions (Eqs. 13 and 14).

$$\frac{\partial \phi}{\partial t} + \nabla \cdot (\mathbf{u}\phi) = \nabla \cdot \left(\frac{\zeta}{\varepsilon^2}\nabla \psi\right) \tag{11}$$

$$\psi = -\nabla \cdot \left(\varepsilon^2 \nabla \phi\right) + \phi\left(\phi^2 - 1\right) \tag{12}$$

$$V_{f,a} = \frac{1+\phi}{2} \tag{13}$$

$$V_{f,l} = \frac{1-\phi}{2} \tag{14}$$

The variables ζ, ξ, and ε are mobility, mixing energy density, and the interface thickness, respectively. The mobility is assumed to be $50\varepsilon^2$, and the mixing energy density is equal to $3\varepsilon\sigma/\left(2\sqrt{2}\right)$ (where σ is the surface tension of air/water at 20 ºC) while the interface thickness is half the maximum element size. The surface tension force (Eq. 15) is then calculated from the phase field by multiplying the chemical potential times the gradient of the phase field variable.

$$\mathbf{F}_{ST} = \left(\frac{\xi}{\varepsilon^2}\psi\right)\nabla\phi \tag{15}$$

Mass Transfer – Diffusion, Convection and Reaction

To track the dispersed phases of calcium ions, nanoclay particles, unbound binding sites, and bound complexes (calcium ions with nanoclay), a diffusion-convection-reaction equation (Eq. 16) was used (*i* represents ion, nanoclay particles, unbound sites and bound ion-nanoclay complexes). The binding site concentration is expressed as equivalent (eq) of hydrogen per m^3. The $CaCl_2$ is in units of mol m^{-3}. The diffusivity was based on literature for the calcium chloride (7.54×10^{-10} m^2 s^{-1} [6]) ions and the diffusivity of other species was set to zero because they consisted of large bound molecules with negligible diffusivity.

$$\frac{\partial c_i}{\partial t} + \nabla \cdot (\mathbf{u}_c c_i) = \nabla \cdot (D \nabla c_i) + R_i \qquad (16)$$

$$\mathbf{u}_c = \mathbf{u} + \mathbf{u}_{slip} + \left(6\frac{D}{\varepsilon} + 6|\mathbf{u}|\right) \underbrace{\left(\frac{\nabla\phi}{|\nabla\phi|}\right)}_{\text{interface normal}} \underbrace{\left(V_{f,a}\left(1 - V_{f,a}\right)\right)}_{\text{location of interface}} \qquad (17)$$

$$\mathbf{u}_{slip} = \frac{\left(\rho_f - \rho_{clay}\right)\hat{d}_p^2}{18\mu_f}\left(-\frac{d\mathbf{u}}{dt} - \mathbf{u} \cdot \nabla\mathbf{u} + \mathbf{g}\right) \qquad (18)$$

$$\hat{d}_p = d_p \, {}^{\mu_p}\!/_{\mu_w} \qquad (19)$$

The velocity, $\mathbf{u}_c$ (Eq. 17) was set to a combination of the fluid velocity as calculated by Navier-Stokes, a slip velocity $\mathbf{u}_{slip}$ (Eq. 18), and a corrected velocity for near interface particles. The nanoclay particle diameter used for the slip velocity, $\hat{d}_p$, was equal to the ratio of the solution viscosity to the viscosity of water times the nanoclay particle diameter. Thus, as individual particles bonded (increasing the nanoclay viscosity), the effective particle diameter would increase. The diffusivity divided by the interface thickness gives a counter velocity to molecules trying to diffuse across the interface. The absolute value of the fluid velocity times the gradient of the phase field normalized by the absolute value itself, creates a normal, opposing velocity at the interface only. The volume fraction of air multiplied by one minus itself is zero everywhere except at the interface. Hence, the corrected velocity adds an equal counter-diffusive and convective flux only at the interface.

The reaction rate (Eqs. 20-23) is assumed to be second order with a constant rate constant. A sensitivity analysis of the rate-constant assumption was performed until the simulation matched the experiment. Thus, the generation of bound ion-nanoclay matrix is not formed instantaneously but gradually occurs. Calcium has a 2^+ charge and one

calcium ion binds to two sites on the nanoclay. The number of binding sites for the nanoclay is based on the cation exchange capacity (CEC) which is assumed to be 7.9×10^{-1} eq kg^{-1} [7].

$$R_{ion} = -kc_{ion}c_{unbound}(1 - V_{f,a}) \tag{20}$$

$$R_{clay} = 0 \tag{21}$$

$$R_{unbound} = -2kc_{ion}c_{unbound}(1 - V_{f,a}) \tag{22}$$

$$R_{bound} = 2kc_{ion}c_{unbound}(1 - V_{f,a}) \tag{23}$$

The total number of binding sites, unbound sites and bound sites is conserved ($c_{bound} = c_{clay}\rho_l CEC - c_{unbound}$). The reaction rate constant was 1 m^3 mol^{-1} s^{-1}.

Boundary and Initial Conditions

The boundary conditions are no slip and no flux everywhere with a reference point at the top right as the pressure constraint. The initial velocity is zero everywhere except the droplet has an initial downward velocity, *v*, which was ranged from 0.3 to 1.7 m s^{-1}.

Initially, there is 2 weight percentage of nanoclay particles with an initial concentration of binding sites, *C*, in the droplet. The initial bound concentration of Ca^{2+} was either 0.5, 1, or 2 mol m^{-3} to vary the initial droplet viscosity.

Numerical Implementation

The equations are solved using a commercial finite element package, COMSOL Multiphysics version 5.0 (COMSOL Multiphysics Burlington, MA). The COMSOL modules, *Laminar Two-Phase Flow-Phase Field* and *Transport of Diluted Species*, were used to solve the governing equations for fluid flow (Eqs. 1 and 2) and phase (Eq.

11), and the dispersed phases (Eq. 16). The mesh was 145072 triangular elements with a maximum size of 50 µm. The relative and absolute tolerances were set to 1×10^{-3} and 5×10^{-4} respectively. A backward differentiation formulas stepping algorithm was used with a maximum time step of 10 µs thereafter. A PARDISO solver was used with constant damping. The computational run time was approximately three days on a computer with 32 GB of RAM and a 2.0 GHz dual core Intel[R] Xeon CPU E5-2620 processor.

Simulation Results

Supplementary Table 1 shows the Dahmköhler numbers, *Da*, of all five shapes. There are three important ranges: a high (above 0.03), a medium (0.015-0.03), and a low (below 0.015) range. In the high range, the reaction rate dominates over fresh interface rate leading to the nanoclay quickly bonding and forming compact teardrop shape. In the low range, the nanoclay does not react quickly enough and the rate of new interface dominates leading to a fractured structure. While in the middle range, neither force dominates and the nanoclay reacts on a time scale similar to the interfacial creation rate such that complex structures can form. The cap has a high Dahmköhler number preventing the stalk, observed in the jellyfish, from forming.

Shapes

Figure 3c in the main text shows the polymer viscosity with time while Supplementary Fig. 7 shows the corresponding ion concentration based on the effect of varying the initial velocity and viscosity. The model results show how each of the five shapes form. For low viscosity shapes, such as the donut shape, the nanoclay follows

the fluid field creating the vortices needed. As the viscosity is increased, the slip velocity increases and the teardrop, jellyfish, and cap are formed. While at sufficiently high velocities, the nanoclay is dispersed regardless of the viscosity and is fractured.

The teardrop and jellyfish share a characteristic feature, the stalk along the centerline. The stalk forms for two reasons: lower ion concentration along the centerline and less nanoclay bound to ions.

The lower ion concentration (shown in blue) reduces the reaction rate and hence the viscosity. The less nanoclay bound to ions leads to a lower slip velocity meaning the nanoclay follows the fluid velocity field more. Instead of the nanoclay sinking, it follows the fluid field upwards toward the air-water interface, generating the stalk.

The cap does not exhibit a stalk since the nanoclay is spread out upon impact, reacting quickly and thus sinking. Whereas the teardrop creates a moving front that has reacted with most of the ions leading a lag in the reaction at the stalk since ions are depleted.

The fracture is simply formed by the extremely high shear stress dispersing the nanoclay such that it cannot react.

Average Shear Rate with Time

Supplementary Fig. 8 shows the weighted average shear rate with time of the nanoclay drop. The two noticeable features are the initial high shear rate peak followed by a possible second peak. The second peak represents the off-center vortices that makes the jellyfish and donut shape. The fracture has much higher peaks than all the other shapes. The teardrop and cap do not have a secondary vortices after impact

because they have fully reacted stopping flow.

Nomenclature

English symbols

a	interfacial area, m^2
c	concentration, mol m^{-3}
C	initial binding site concentration, mol m^{-3}
CEC	cation exchange capacity, eq kg^{-1}
d_p	nanoclay particle diameter, m
$\hat{d}_p$	equivalent nanoclay particle diameter, m
D	diffusivity m^2 s^{-1}
Da	Damköhler
e	strain tensor, s^{-1}
F_{ST}	surface tension force, N m^{-3}
g	gravity m s^{-2}
I	identity matrix
p	pressure, Pa
u	velocity, m s^{-1}
Oh	Ohnesorge
R	reaction rate mol m^{-3} s^{-1}
Re	Reynolds number
t	time, s
v	initial velocity, m s^{-1}

V_f	fluid volume fraction
We	Weber number

Greek symbols

μ	dynamic viscosity, Pa s
σ	surface tension
σ_u	surface tension
ρ	density, kg m-3
γ	shear rate s-1
ζ	mobility, m^2
ξ	mixing energy density,
ϕ	phase field variable
ε	interface thickness, m
ψ	chemical potential
υ_{clay}	clay volume fraction

Subscripts

a	air
bound	bound binding sites
clay	nanoclay
f	fraction
ion	ion
l	liquid

p	polymer
slip	slip velocity
ST	surface tension
unbound	unbound binding sites
0	initial

Supplementary Note 5

Detailed discussion of the formation and shape control of the VRP

The ability to freeze the VRP into different shapes arises from the balance between crosslinking and convective mixing of the droplet and bath liquids, where convection results from the droplet's impact inertia. If droplet inertia is too great upon impact, surface tension cannot hold the droplet together (for any shape) and fracture occurs (to the right of the vertical line at We=45.7 in figure 3b). In this case, the drop breaks up immediately upon impact and the resulting particle pieces reflect the shapes of the broken bits.

Evolution of the shapes formed by the nanoclay after impact are illustrated in Figure 1a. Vorticity is generated at the interface between the droplet and the liquid pool. This diffusion of vorticity leads to mixing between the bath and the droplet solutions. The different shapes are formed depending on the amount of vorticity diffused into the droplet. The teardrop shape occurs first representing the least amount of diffused vorticity and the donut shape last representing the most diffused vorticity. Intermediate are the jellyfish and cap shapes.

When the droplet brings reactant A (drop solution) into contact with reactant B (bath solution), A and B react. If mixing occurs sufficiently slowly relative to reaction (large Da in figure 3b), a shape from early in the evolutionary sequence of shapes becomes the VRP. Note that increasing the droplet viscosity relative to the bath viscosity (viscosity contrast) effectively slows mixing regardless of impact inertia. For this reason, teardrop-VRP are favored by high viscosity contrast and high Da (figure 3b).

As convective mixing is increased by lowering the droplet viscosity, the *Da* decreases and cap-VRP are observed. Further increases in the mixing rate (all else equal) leads to jellyfish- and donut-VRP, respectively.

Alternatively, Figure 3b could be read at a constant viscosity ratio with increasing *We*. Increasing *We* represents an increase in the impact inertia of the droplet, and thus an increase in the rate of mixing of the droplet within the bath at any given time. Therefore, an increase in *We* with constant reaction rate leads to a decrease in *Da* and the same progression of VRP shapes is observed.

Interesting is the reversal of gelation of the jellyfish- and cap- shapes with decreasing *Da* numbers. Without reaction, the time progression of the shapes is teardrop-, jellyfish-, cap- and donut-. Whereas with reaction decreasing *Da* forms the shapes in the order teardrop-, cap-, jellyfish- and donut-. Further discussion on this observation, however, is outside the scope of this work.

Supplementary Note 6

Theory for Damköhler number isocontours in Figure 3b

Based on experimental and simulation results, isocontours of the Damköhler number, Da, were overlayed that create boundaries between each structure. It was experimentally observed that a log-log plot of the water, *w*, Reynolds number (Eq. 24) multiplied by the square root of the viscosity ratio ($\frac{\mu_w}{\mu_d}$ where *d* represents droplet) versus the droplet Weber number (Eq. 25) multiplied by the viscosity ratio created a straight line (Eq. 26 with R^2 = 0.9945):

$$\mathrm{Re}_w = \frac{\rho_w v r}{\mu_w} \tag{24}$$

$$\mathrm{We}_d = \frac{\rho_d v^2 r}{\sigma_{da}} \tag{25}$$

$$\log_{10}\left(\mathrm{We}_d\left(\frac{\mu_w}{\mu_d}\right)\right) = 2.2\log_{10}\left(\mathrm{Re}_w\left(\frac{\mu_w}{\mu_d}\right)^{0.5}\right) - 5.8 \tag{26}$$

where ρ, μ, σ, r, and v are the density, dynamic viscosity, surface tension, droplet radius, and velocity.

Thus, the droplet Weber number, water Reynolds number, and viscosity ratio are a function (Eq. 27) of one another.

$$\mathrm{We}_d\left(\frac{\mu_w}{\mu_d}\right) = 10^{-5.8}\left[\mathrm{Re}_w\left(\frac{\mu_w}{\mu_d}\right)^{0.5}\right]^{2.2} \tag{27}$$

The experimental Damköhler number (Eq. 28) was estimated based on the simulation reaction rate (kC=16.2 s^{-1}) divided by an intuitive, theoretical shear rate (Eq. 29).

$$\mathrm{Da} = \frac{kC}{\gamma} \tag{28}$$

$$\gamma = \frac{v}{rf\left(\frac{\mu_w}{\mu_d}\right)} \tag{29}$$

An assumption was made that the unknown viscosity function, *f*, was equal to the viscosity ratio (Eq. 30) raised to an unknown exponent, *n*. This assumption is based on the fact that as the droplet viscosity increased, the shear within the droplet would decrease upon impact. Hence favoring a lower shear rate and a higher Damköhler number.

$$f\left(\frac{\mu_w}{\mu_d}\right) = \left(\frac{\mu_w}{\mu_d}\right)^n \tag{30}$$

Using 1.5 mm as the radius, r, and 10^{-6} m^2/s for water's kinematic viscosity, the Damköhler number thus equals:

$$\mathrm{Da} = \frac{kCr}{v}\left(\frac{\mu_w}{\mu_d}\right)^{-n} \tag{31}$$

$$\begin{aligned} \mathrm{Da} &= \frac{kCr}{v}\left(\frac{\rho_w r \mu_w}{\rho_w r \mu_w}\right)\left(\frac{\mu_w}{\mu_d}\right)^{-n} \\ &= \frac{kC\rho_w r^2}{\mu_w}\left(\frac{\mu_w}{v\rho_w r}\right)\left(\frac{\mu_w}{\mu_d}\right)^{-n} \\ &\approx 36\,\mathrm{Re}_w^{-1}\left(\frac{\mu_w}{\mu_d}\right)^{-n} \end{aligned} \tag{32}$$

such that the Damköhler and Reynolds number and viscosity function equals a constant:

$$\mathrm{Da}\,\mathrm{Re}_w\left(\frac{\mu_w}{\mu_d}\right)^n \approx 36 \tag{33}$$

Substituting Eq. (33) into Eq. (27) for the Reynolds number yields:

$$\begin{aligned} \mathrm{We}_d\left(\frac{\mu_w}{\mu_d}\right) &= 10^{-5.8}\left[36\mathrm{Da}^{-1}\left(\frac{\mu_w}{\mu_d}\right)^{-n}\left(\frac{\mu_w}{\mu_d}\right)^{0.5}\right]^{2.2} \\ \left[10^{5.8}\,\mathrm{We}_d\left(\frac{\mu_w}{\mu_d}\right)\right]^{1/2.2} &= 36\mathrm{Da}^{-1}\left(\frac{\mu_w}{\mu_d}\right)^{-n+0.5} \\ \mathrm{Da} &= 36\left(10^{-2.64}\right)\left(\frac{\mu_w}{\mu_d}\right)^{-n+0.5-0.45}\mathrm{We}_d^{-1/2.2} \\ \mathrm{Da} &\approx 36\left(10^{-2.64}\right)\left(\frac{\mu_w}{\mu_d}\right)^{-n}\mathrm{We}_d^{-1/2} \end{aligned} \tag{34}$$

By then plotting Damköhler isocontours for different values of *n* on the experimental data, a value of 0.5 fit best in terms of segregating shapes. Furthermore, comparing the final form of Eq. (34) with Eq. (32) leads to the fact that the water Ohnesorge number, *Oh*, is approximately the $10^{-2.64}$ coefficient. Thus the Damköhler number is a

dimensionless reaction times the ratio of viscous and inertial and viscous forces (Ohnesorge number), divided by the intertial time scale (in the form of the Weber number), and scaled by the viscosity ratio.

$$\mathrm{Da} \approx \frac{36\left(10^{-2.64}\right)}{\mathrm{We}_d^{1/2}\left(\frac{\mu_w}{\mu_d}\right)^{0.5}} \approx \frac{\left(\frac{kC\rho_w r^2}{\mu_w}\right)\mathrm{Oh}_w}{\mathrm{We}_d^{1/2}\left(\frac{\mu_w}{\mu_d}\right)^{0.5}} \tag{35}$$

Supplementary Note 7

Ion-competing buffer for alginate and chitosan hydrogel microVRP

Our first trial to decrease the alginate gelation speed is to decrease the concentration of the crosslinking buffer. As shown in Supplementary Table 2, while decreasing the $CaCl_2$ concentration, there was indeed a tendency that the gelation speed was slowed down. When the $CaCl_2$ concentration was 10 mM, jellyfish-microVRP started to form instead of teardrop-microVRP formed at a higher concentration. However, we could not obtain other microVRP such as cap- or donut-ones by decreasing the buffer concentration since the low concentration ion buffer cannot completely crosslink the hydrogel, resulting in fractures. Then, we developed a novel ion-competing buffer containing both a primary crosslinking agent ($CaCl_2$) and a competing agent ($MgCl_2$). Mg^{2+} resembles Ca^{2+} but cannot crosslink the alginate by itself. When the alginate drop enters the ion-competing buffer, the Mg^{2+} will compete with the $CaCl_2$ by occupying the reaction sites, thus slow down the reaction speed. By using this ion-competing buffer, we were able to fabricate cap-microVRP and donut-microVRP. We implemented a similar strategy to chitosan and achieved mass production of chitosan donut-microVRP (Supplementary Table 3) as well.

Supplementary Methods

Rat Islet Isolation and Purification. Sprague-Dawley rats from Charles River Laboratories weighing approximately 300 grams were used for harvesting islets. All rats were anesthetized using 3% isofluorane in oxygen and maintained at the same rate throughout the procedure. Isolation surgeries were performed as described by Lacy and Kostianovsky[8]. Briefly, the bile duct was cannulated and the pancreas was distended by an in vivo injection of 0.15% Liberase (Research Grade, Roche) in RPMI 1640 media solution. The pancreas was digested a 37 °C water bath for 30 minutes. The digestion was stopped by adding 10−15 mL of cold M199 media with 10% heat-inactivated fetal bovine serum and a slight shaking. Digested pancreases were washed twice in the same aforementioned M199 media, filtered through a 450 µm sieve, and then suspended in a Histopaque 1077 (Sigma)/M199 media gradient and centrifuged at 1,700 RCF at 4 °C. Depending on the thickness of the islet layer that was formed within the gradient, this step was repeated for higher purity islets. Finally, the islets were collected from the gradient and further isolated by a series of gravity sedimentations, in which each supernatant was discarded after four minutes of settling. Purified islets were hand-counted by aliquot under a light microscope and then washed three times in sterile 1X phosphate-buffered saline. Islets were then washed once in RPMI 1640 media with 10% heat-inactivated fetal bovine serum and 1% penicillin/streptomycin, and cultured in this medium overnight for further use.

Preparation of Cell-free Lysate. Cell-free lysate was prepared from a BL21 strain of

E. coli. Cells were grown at 37 Celsius in 2x YT media until reaching an OD_{600} of .6. Following this, isopropyl-thiogalactopyranoside (IPTG, 1 mM) was added to the culture media to induce expression of T7 RNA polymerase. Once the culture reached an OD_{600} of 4.5, the cell suspension was washed with a buffer containing 10mM Tris-acetate (pH 8.2), 60 mM potassium glutamate, 14mM magnesium acetate, and 1mM dithriothreitol (DTT) with /0.5% 2-mercaptoethanol (2-ME). The suspension was then pelleted and re-suspended in the same buffer solution but without 2-ME. Following this, cells were ruptured using a French Press, centrifuged at 12,000 RCF for 10min and incubated for 30 minutes at 37 Celsius. The cell-free lysate used for protein expression contained 27% E. coli extract which was supplemented with 57 mM Hepes-KOH (pH 8.2), 1.2 mM ATP, 2 mM DTT, 0.17 mg ml^{-1} E. coli total tRNA, 1.2 mM ATP, 0.85 mM each of CTP, GTP and UTP, 0.64 mM cAMP, 90 mM potassium glutamate, 80 mM ammonium acetate, 12 mM magnesium acetate, 34 mg ml^{-1} folinic acid, 1.5 mM each of 20 amino acids, 2% PEG (8000), 67 mM creatine phosphate and 3.2 mg ml^{-1} creatine kinase. This solution was aliquoted and stored at -80 Celsius.

Supplementary References

1. Walker TW, Logia AN, Fuller GG. Multiphase flow of miscible liquids: jets and drops. *Exp. Fluids* **56**, 14 (2015).

2. Ahmadlouydarab M, Feng JJ. Motion and coalescence of sessile drops driven by subtrate wetting gradient and external flow. *J. Fluid Mech.* **746**, 214-235 (2014).

3. Dong S. On imposing dynamic contact-angle boundary conditions for wall-bounded liquid-gas flows. *Comput. Meth. Appl. Mech. Eng.* **247**, 179-200 (2012).

4. Pereira AS, Pinho FT. Turbulent pipe flow of thixotropic fluids. *Int. J. Heat. Fluid Flow* **23**, 36-51 (2002).

5. Cummins HZ. Liquid, glass, gel: The phases of colloidal Laponite. *J. Non-Cryst Solids* **353**, 3891-3905 (2007).

6. Hazel JR, Sidell BD. A method for the determination of diffusion-coefficients for small molecules in aqueous-solution. *Anal. Biochem.* **166**, 335-341 (1987).

7. Thomas PC, Cipriano BH, Raghavan SR. Nanoparticle-crosslinked hydrogels as a class of efficient materials for separation and ion exchange. *Soft Matter* **7**, 8192-8197 (2011).

8. Lacy PE, Kostiano.M. Method for isolation of intact islets of Langerhans from rat pancreas. *Diabetes* **16**, 35-39 (1967).
